# Modeling children’s moral development in postwar Taiwan through naturalistic observations preserved in historical texts

**DOI:** 10.1038/s41598-024-59985-6

**Published:** 2024-04-21

**Authors:** Zhining Sui, Qinyan Wang, Jing Xu

**Affiliations:** 1 Department of Biostatistics, University of Washington, 1410 NE Campus Parkway, Seattle, WA 98195 USA; 2 Department of Biostatistics and Computational Biology, University of Rochester, 265 Crittenden Boulevard, Rochester, NY 14642 USA; 3 Department of Linguistics, University of Washington, 1410 NE Campus Parkway, Seattle, WA 98195 USA; 4 Amazon.com, Inc., 400 9th Ave N, Seattle, WA 98109 USA; 5 Department of Anthropology and eScience Institute, University of Washington, 1410 NE Campus Parkway, Seattle, WA 98195 USA

**Keywords:** Psychology, Human behaviour

## Abstract

A core issue in the interdisciplinary study of human morality is its ontogeny in diverse cultures, but systematic, naturalistic data in specific cultural contexts are rare to find. This study conducts a novel analysis of 213 children’s socio-moral behavior in a historical, non-Western, rural setting, based on a unique dataset of naturalistic observations from the first field research on Han Chinese children. Using multilevel multinomial modeling, we examined a range of proactive behaviors in 0-to-12-year-old children’s peer cooperation and conflict in an entire community in postwar Taiwan. We modeled the effects of age, sex, kinship, and behavioral roles, and revealed complex interactions between these four variables in shaping children’s moral development. We discovered linkages between coercive and non-coercive behaviors as children strategically negotiated leadership dynamics. We identified connections between prosocial and aggressive behaviors, illuminating the nuances of morality in real life. Our analysis also revealed gendered patterns and age-related trends that deviated from cultural norms and contradicted popular assumptions about Chinese family values. These findings highlight the importance of naturalistic observations in cultural contexts for understanding how we become moral persons. This re-analysis of historically significant fieldnotes also enriches the interdisciplinary study of child development across societies.

## Introduction

The fundamental question of how we become moral persons has intrigued scientists and humanists for centuries. Childhood provides a unique window into human morality and its formation^1–3^. Despite recent progress in tracing the ontogeny of human moral sensibilities, thanks to interdisciplinary dialogues between psychology and anthropology^4,5^, researchers advocate the urgency to broaden our horizons and examine child development in diverse cultural contexts^6^. One reason is that Western-centered sampling biases still persist in developmental science^7^. Another problem is conceptual and methodological biases rooted in different disciplinary traditions: Psychologists approach children as “stubborn autodidacts”^8^ and prioritize standardized experiments over studying the complexity and richness of children’s social life in natural contexts^9^. Cultural anthropologists, on the other hand, tend to view children as “passive assimilators” in their environment^10,11^ and over-emphasize parenting and socialization, rather than children’s active learning^12^.

A promising direction to address these problems is systematic, naturalistic observations in cultural contexts because this approach can produce ecologically valid data on human behavior^13^. Existing observational research has mainly focused on school settings or parent-child interactions in Western, urban communities, therefore studying peer interactions in communal settings in rural, non-Western contexts is imperative^14^. Moreover, examining historical documents can inform the study of human cognition in the past and present^15,16^. Our research is a rare attempt that uses a Bayesian multilevel multinomial logistic model to analyze a significant set of historical texts that documented children’s socio-moral behavior in their everyday lives. These texts are part of what we call “the Wolf Archive,” ethnographic fieldnotes left behind by the renowned anthropologist Arthur Wolf, collected during his first fieldwork in Taiwan (1958–1960). Together with his then wife Margery Wolf, Arthur Wolf conducted the first anthropological research on culturally Chinese children in a village near Taipei at the height of Taiwan’s Martial-law era. Wolf’s original research replicated the Six Cultures Study of Child Socialization (”SCS” thereafter)^17,18^, a landmark project in the history of cross-cultural research^19^. The SCS teamed together anthropologists and psychologists, used a mixed-methods design in fieldwork among communities across six societies, and it has inspired the revival of cross-cultural developmental research today^20^. In particular, the systematic, naturalistic observation called Child Observation remains the SCS’s most enduring legacy^19^. Child Observation in Wolf’s research is of unique value as its methodology improved from the SCS in several aspects: excellent local research assistants, much longer fieldwork, complete household demographic information of the entire village, as well as its observation protocol (see “Methods” section).

We coded these fieldnotes and analyzed a diverse range of behaviors of 213 children below age 13 (calculated by the end of Wolf’s fieldwork), compared to 23 children (ages 3–11) per field site in the SCS. We designed a new behavioral coding system that took inspiration from but also differed from the SCS guide^18^. We combined deductive, top-down and inductive, bottom-up perspectives to better capture the complexity of children’s moral experience in their cultural contexts: Using a top-down approach, we included focal themes in existing literature, e.g., typical prosocial behaviors such as helping (instrumental help), sharing (resources), and comforting (emotional support) and aggressive behaviors such as physical aggression, verbal aggression, etc. Using a bottom-up approach, we added salient themes in local contexts, such as leading, dominating, scolding, tattling, giving a dirty look, requesting for comfort/help/sharing, requesting for access (to play), etc. We targeted this broad list of behaviors (Supplementary Table  S1) as recent theories suggest that human morality consists of multiple types of solutions to problems of cooperation recurrent in human social life, including reducing and resolving conflicts^21^.

Our study aims to understand how demographic factors influence children’s moral development in the cultural contexts of ethnic Han society. We measured individual differences in social behaviors and modeled the effects of age, sex, behavioral roles, and initiator-recipient kinship. We added analysis of recipients, whereas both the SCS and recent research on prosocial or aggressive behavior predominantly focused on initiators^22–24^. We also analyzed the binary variable of initiator-recipient co-residence, as children in this close-knit and high-fertility village often interacted with both siblings and other peers. Notably, most children lived with their biological siblings, in contrast to their parents’ generation when adoption was more common^25^. Finally, we modeled the interactions between different kinds of behaviors, e.g., cooperative and conflictual behaviors, as previous research identified co-development of these behaviors^26^.

The Wolf Archive provides a rare opportunity to examine moral development from infancy to middle childhood (0–12) in an entire community. Recent studies have found that various moral inclinations emerge in early childhood, some in infancy^5,27^, earlier than what classic theories characterized^2,28^. Middle-childhood is also an important phase, as previous research have identified cross-cultural variations^29,30^, more strategic motivations underlying prosocial behavior^31^, and increasing sensitivity to social norms^32,33^. However, age-related changes in prosocial behavior are complex: although a meta-analysis suggests that prosocial behaviors increase as children get older, the results depend on specific study designs and analyses^23^. Our study considers both initiators’ and recipients’ ages in naturally-occurring prosocial behaviors. Studies of aggression prioritize adolescents and elementary school children, as they enter a larger social world and develop more varied aggressive strategies^34^. However, recent studies have shown that physical aggression emerges early in infancy^35,36^. Our study captures the nuances by examining various types of aggression and their age-related trends from infancy to age 12. Also, in this rural community with dense social ties, we consider aggression together with prosocial and other types of behaviors, i.e., dominance, leading, etc., as children’s rich repertoire of strategies to regulate conflicts, facilitate cooperation, and negotiate social statuses^37^.

Gender/sex is another important factor, often examined together with age. We borrowed the terminology in the SCS and our original data to pay respect, using “sex” to refer to children’s biological sex, although we do not presume biological causes of sex-differences in behaviors. Although studies from Western samples showed a general trend of girls being more prosocial than boys^23^ and that such sex differences grew more consistent with age^38^, recent cross-cultural research did not find uniform differences in prosocial behaviors between boys and girls or consistent patterns of gender-age interaction^39^. Research on aggression also revealed complexity: While boys tend to exhibit physical aggression more often than girls^24,40–43^, gender difference in indirect and relational forms of aggression showed mixed results^40^, including in cross-cultural work^41,43^. Ethnographic observations further complicate these patterns, especially considering age-sex interaction^22^. Honoring the SCS’ legacies, our study examined age-sex interaction in prosocial, aggressive, and other behaviors. But going beyond the SCS’ era, we can apply advanced statistical modeling methods to ethnographic data.

This is the first study to systematically examine the social behavior of an entire community of culturally Chinese children. Arthur and Margery Wolf’s previous research from this community helped establish the foundations for studying the traditional Chinese family^25,44–46^. However, children are not a focus in the study of Chinese families, despite the fact that childhood experience is critical in shaping core features of traditional Chinese families, such as gender biases and inequality^47^. Even Margery Wolf’s famous article on child-training^45^ prioritized socialization values, and children existed passively, as an object of childrearing ideologies and in the shadow of parent-child ties. Therefore, our re-analysis not only can bring to light the obscured world of children’s social life, especially their peer interactions, but also examine the relationship between cultural values and behavioral reality.

In this regard, we will address three focal questions after comprehensively modeling all the behavioral data we coded: First, children’s leadership dynamics: How do children mobilize themselves into group activities, enact norms, impart moral knowledge, or establish authority in the process? The Wolfs’ works hardly ever examined this topic, but we found it ubiquitous in children’s everyday play, therefore including behaviors such as leading (non-coercive) and scolding (moral criticism) into our coding scheme, in addition to dominating (coercive) examined in the scarce observational research on Chinese children^48^. Second, age-related trends: Margery Wolf noted that, in this traditional Chinese community where age is an important factor in social hierarchy, caregivers used harsher discipline on older children but a more lenient approach towards younger children, as younger children were assumed to have little capacity for moral reasoning^45^. Do age-related behavioral trends conform to entrenched cultural expectations in Chinese societies from the past to the present, that older children become role models for younger children and yield to them during conflicts?^45,49^ Finally, sex-differences: Are sex-differences in behaviors aligned with ascribed gender roles according to moral precepts, i.e., submissive girls? Margery Wolf’s classic ethnography based on this community has highlighted adult women’s agency despite their structurally subordinate position in the patrilineal, patriarchal Han Chinese family^50^. However, girls seemed docile and passive in her limited exploration of children’s world. Systematically examining behaviors in conflict situations might reveal patterns that diverge from these impressionistic observations. Taken together, our study highlights children’s agency in their self-organized social world, in contrast to the “passive-child” imagery rooted in earlier paradigms of the Chinese family.

Six decades later, the once village is now part of New Taipei City and it is impossible to replicate such systematic observations of children’s communal life^51^. Childhood in Taiwan, China, and East Asia more broadly has experienced profound changes as a result of rapid economic development, urbanization, and industrialization, together with fertility decline and transformation of family structures and values^52,53^. This re-analysis of old fieldnotes provides a rare reference to compare and contrast with contemporary East Asian childhoods, enriching the interdisciplinary study of child development in cultural contexts.

## Results

### Descriptive statistics

Demographic variables in our study include the initiator’s age and sex, the recipient’s age and sex, and household numbers of initiators and recipients. Our sample includes 213 children from 70 households: 102 girls (mean age at the study’s outset = 4.23 years, SD = 3.01), 98 boys (mean age at the study’s outset = 4.55 years, SD = 2.72), plus 13 infants born during the 2-year fieldwork period (7 girls and 6 boys). On average, each child contributed 61.10 behavioral occurrences (SD = 52.08), each household 185.91 occurrences (SD = 144.14). Boys participated more in observed behaviors than girls, both as initiators and recipients (Supplementary Fig.  S1). The number of children per household varied from 1 to 9 (mean = 3.04 children, SD = 1.80): 32 households had more girls, 27 had more boys, and 11 had an equal distribution (Supplementary Fig.  S2).

**Figure 1:**
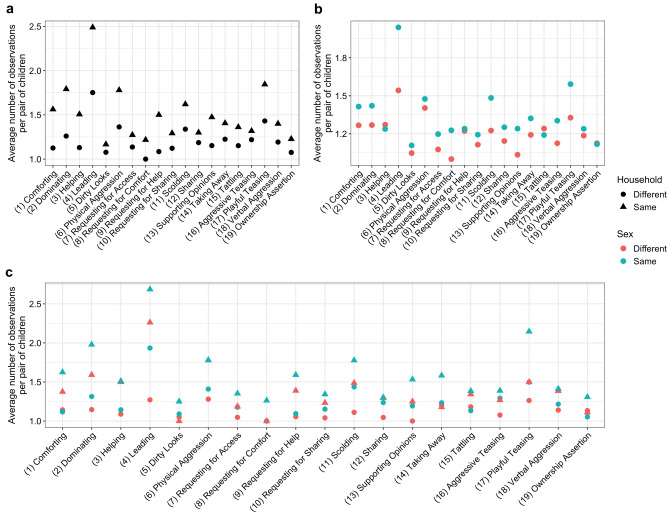
Average number of observations per pair of children for each behavior (i.e., total number of observations/total number of unique pairs). (**a**) Comparison between children pairs from the same or different households. (**b**) Comparison between children pairs with the same or different sexes. (**c**) Comparison between children pairs from the same or different households, and of the same or different sexes. Exact cross-sex variations with sexes specified are shown in Supplementary Fig.  S3.

Overall, *leading* emerged as the predominant behavior across children, irrespective of household status, and behaviors happened more frequently among children from the same household, except for *comforting* and *requesting for comfort* (Supplementary Fig.  S1). Comforting behavior typically occurs when a younger sibling seeks comfort from an older sibling through crying or whining. The unexpectedly higher number of observations of comforting behavior between children from different households arose from a few outlier dyads. Upon accounting for the number of unique pairs displaying each behavior, it became evident that the average number of observations per dyad (except for *dirty looks*) was consistently higher when both children in the interaction were from the same household (Fig.  1b). Furthermore, same-sex dyads interacted more frequently than cross-sex dyads, except for *helping*, *tattling*, and *ownership assertion* (Fig.  1a). we also discovered initiator-recipient variations based on sex and household (Fig.  1c).

Finally, we discovered significant variations in the average age of initiators and recipients for behaviors like *comforting* and *requesting for comfort* (Supplementary Fig.  S4), which can be explained by the typical sibling interactions. However, for behaviors such as *requesting for sharing* and *supporting opinions*, the difference in average age-at-observation between initiators and recipients was relatively small as these behaviors often happened between same-age peers.

### Comparing models

We fitted four Multilevel Multinomial Behavioral Models^54^. Each of the models, Model_i, Model_iF, Model_ih, and Model_ihF, has different components (see “Methods” section). Model_ihF, including all the components, showed the highest level of support based on the Watanabe-Akaike information criterion (WAIC) comparison^55^ (Table 1). The probability that this model will make the best predictions on new data relative to the other three models is 74.9%. Model_iF had 25.1% of the model weight, indicating that the inclusion of fixed effects improved the predictive performance. The inclusion of household-level random effects has a limited impact on the model’s performance. This finding is consistent with the overlapping standard deviation observed for Model_i and Model_ih and for Model_iF and Model_ihF (Supplementary Fig.  S5). Therefore we will mainly focus on the discussion of Model_i and Model_iF. Results from Model_ih and Model_ihF are included in Supplementary Information.

**Table 1:** Comparison of WAIC, $\Delta$WAIC, and weights for four models.

Model	Random effect	Predictor variables	WAIC ($\Delta$WAIC)	Weight
Model_i	Individual-level	None	33,229.93 (388.30)	< 0.001
Model_ih	Individual-level + Household-level	None	33,239.64 (398.01)	< 0.001
Model_iF	Individual-level	Age + sex + age $\times$ sex + household status	32,843.83 (2.19)	0.251
Model_ihF	Individual-level + Household-level	Age + sex + age $\times$ sex + household status	32,841.64 (ref)	0.749

Models were named based on the effects they included, where “i” and “h” stand for individual-level and household-level random effects, respectively, and “F” stands for fixed effects.

### Individual variations in behaviors

Model_i includes only the intercept and individual random effects for initiators and recipients. We did not focus on the intercept coefficients because the predicted probabilities closely matched the corresponding percentages from the empirical data (Supplementary Fig.  S6). The extent of individual variation in exhibiting each behavior differed between initiators and recipients (Table 2). Several behaviors had relatively low variances in the initiator’s random effects, such as *requesting for sharing* and *supporting opinions*, suggesting that the probabilities of initiating these behaviors did not vary greatly among children. On the other hand, *requesting for comfort* had a notably higher variance in the initiator’s random effect, suggesting that a subset of children, especially the younger ones, were more likely to initiate this behavior. Moreover, there was a distinctively high variance in the recipient’s random effect for *comforting*. This implies that some children were more likely to receive voluntary comfort from their peers.

Model_iF included additional fixed effects from sex, age, and kinship of initiators and recipients based on Model_i, giving smaller variance estimates of the initiators’ random effects as compared to the variance estimates obtained in Model_i for almost all the behaviors (Table 2). This implies that the predictor variables accounted for the substantial individual-level variance of initiators in all behaviors, except for *helping*. Similarly, the recipients’ individual variations discovered in Model_i can be substantially explained by these predictor variables in all behaviors other than *leading* and *taking*.

**Table 2:** Variance estimates of the individual random effects in Model_i and Model_iF.

		Initiator		Recipient	
		Model_i	Model_iF	Model_i	Model_iF
(1)	Comforting	0.61 (0.26)	0.22 (0.17)	2.07 (0.27)	0.42 (0.26)
(2)	Dominating	0.44 (0.08)	0.25 (0.10)	0.39 (0.08)	0.15 (0.09)
(3)	Helping	0.37 (0.14)	0.39 (0.13)	0.26 (0.13)	0.24 (0.13)
(4)	Leading	0.46 (0.07)	0.28 (0.06)	0.26 (0.07)	0.27 (0.06)
(5)	Look	0.69 (0.27)	0.31 (0.21)	0.21 (0.16)	0.20 (0.15)
(6)	Physical aggression	0.50 (0.09)	0.37 (0.10)	0.40 (0.09)	0.23 (0.11)
(7)	Requesting for access	0.59 (0.12)	0.55 (0.12)	0.82 (0.13)	0.61 (0.12)
(8)	Requesting for comfort	2.68 (0.40)	0.42 (0.29)	0.39 (0.30)	0.28 (0.21)
(9)	Requesting for help	0.83 (0.19)	0.38 (0.21)	0.58 (0.20)	0.28 (0.18)
(10)	Requesting for sharing	0.29 (0.14)	0.24 (0.13)	0.26 (0.14)	0.26 (0.14)
(11)	Scolding	0.50 (0.08)	0.15 (0.09)	0.20 (0.10)	0.16 (0.09)
(12)	Sharing	0.36 (0.14)	0.36 (0.14)	0.47 (0.12)	0.46 (0.11)
(13)	Supporting opinions	0.33 (0.18)	0.30 (0.17)	0.73 (0.14)	0.63 (0.14)
(14)	Taking	0.40 (0.15)	0.40 (0.14)	0.17 (0.11)	0.19 (0.12)
(15)	Tattling	0.58 (0.13)	0.44 (0.15)	0.29 (0.15)	0.26 (0.14)
(16)	Teasing (aggressive)	0.46 (0.10)	0.35 (0.11)	0.28 (0.13)	0.22 (0.13)
(17)	Teasing (playful)	0.38 (0.08)	0.33 (0.08)	0.32 (0.08)	0.30 (0.08)
(18)	Verbal aggression	0.75 (0.12)	0.57 (0.12)	0.34 (0.16)	0.29 (0.15)

The reported quantities are the standard deviations of the random effects, while the values in parentheses are the standard deviations of these quantities in the posterior samples.

### Correlations between different behaviors

Model_i revealed correlations among individual random effects across behaviors for both initiators and recipients (Supplementary Table  S2), indicating how different behaviors are linked together for the same children. Note that all the probabilities reported in the following paragraphs are the relative probabilities as compared to the reference behavior, *Ownership Assertion*.

**Figure 2:**
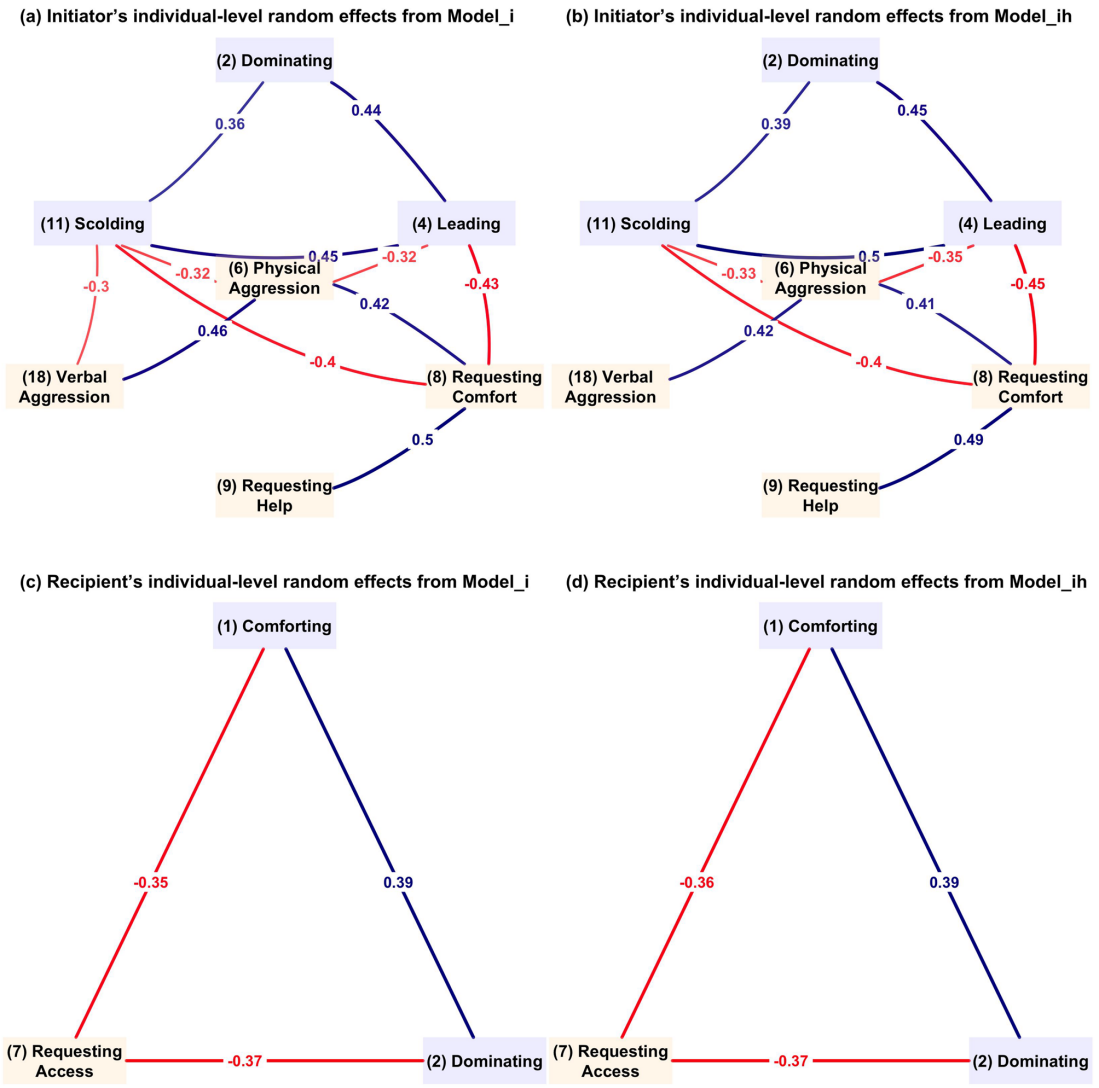
Significant correlation of initiator’s and recipient’s individual random effects across behaviors estimated by Model_i and Model_ih.

For initiators, correlations among seven behaviors were statistically significant. We partitioned them into two groups (Fig. 2), discovering positive correlations within each group but negative correlations across the two: one group consists of *dominating*, *leading*, *scolding*, and the other includes *physical aggression*, *requesting for comfort*, *requesting for help*, and *verbal aggression*. Within the first group of behaviors, children who were more likely to initiate leading behaviors also showed a propensity for dominating ($\rho _{4,2}=0.438$) and scolding ($\rho _{11,4}=0.451$), and dominating others was positively correlated with scolding ($\rho _{11,2}=0.358$). For behaviors in the second group, children prone to initiating physical aggression also resort to verbal insults when provoked ($\rho _{18,6}=0.456$). Notably, we also discovered a counter-intuitive positive correlation between initiating physical aggression and requesting comfort ($\rho _{8,6}=0.422$), highlighting the simultaneous occurrence of anti-social and prosocial behaviors among the same children. Besides, a positive correlation between *requesting comfort* and *requesting help* ($\rho _{9,8}=0.501$) among the same initiators reflects a connection between the expression of instrumental and emotional needs. Behaviors across the two groups were negatively correlated. For the same children, the probability of leading was negatively correlated with physical aggression ($\rho _{6,4}=-0.321$) and requesting comfort ($\rho _{8,4}=-0.426$). Scolding was negatively correlated with requesting comfort ($\rho _{11,8}=-0.402$) and initiating aggression, both physically ($\rho _{11,6}=-0.322$) and verbally ($\rho _{18,11}=-0.297$).

For the same recipients, correlations among three behaviors were statistically significant: *comforting*, *dominating*, and *requesting access* (Fig. 2). The probability of a child receiving requests for access was negatively correlated with that of being comforted ($\rho _{1,7}=-0.351$) or dominated ($\rho _{2,7}=-0.370$). On the other hand, children who were frequently targeted for dominance were also more likely to receive comfort from others ($\rho _{1,2}=0.393$).

### Demographic effects

Model_iF included several predictor variables in addition to Model_i. Compared to the variance estimates obtained in Model_i, the variance estimates of the initiators’ random effects decreased for almost all the behaviors (Table 2), indicating that individual-level variance of initiators discovered in Model_i can be substantially explained by sex, age, and kinship for all behaviors except *helping*. Similarly, the predictor variables accounted for substantial individual-level variance among recipients in all behaviors other than *leading* and *taking*. We predicted the probabilities of each of the 19 behaviors between an “average” recipient and an “average” initiator based on demographic variables (age, sex, and household status/proxy for kinship), using the estimated coefficients obtained from Model_iF.

**Table 3:** The trends of predicted probabilities of 19 behaviors with increasing ages of initiator and recipient with different sexes, when the initiators and the recipients were from the same household (the trends for children from different households are summarized in Supplementary Table  S8).

		Increasing initiator’s age		Increasing recipient’s age	
		Female initiator	Male initiator	Female recipient	Male recipient
(1)	Comforting	Increasing	Increasing	Decreasing	Decreasing
(2)	Dominating	Increasing	Increasing	(1,2)	Decreasing
(3)	Helping	8	(8,10)	[3,4]	[4,6]
(4)	Leading	Increasing	Increasing	(4,5)	(3,5)
(5)	Dirty Looks	[3,4]	Decreasing	Increasing	Increasing
(6)	Physical aggression	[2,3)	(1,2)	[2,3]	Decreasing
(7)	Requesting for access	2	Decreasing	Increasing	Increasing
(8)	Requesting for comfort	Decreasing	Decreasing	Increasing	Increasing
(9)	Requesting for help	Decreasing	Decreasing	Increasing	Increasing
(10)	Requesting for sharing	[2,3)	(3,5)	10	Increasing
(11)	Scolding	Increasing	Increasing	(9,10)	(9,10)
(12)	Sharing	5	(1,2)	(2,3)	Increasing
(13)	Supporting opinions	[3,4]	Increasing	Increasing	Increasing
(14)	Taking Away	[2,3]	(2,3)	(5,6)	(8,9)
(15)	Tattling	[6,7]	[0,1]	[2,3)	Increasing
(16)	Aggressive teasing	(2,3)	(3,5)	[4,5]	Decreasing
(17)	Playful teasing	5	Increasing	[9,10]	[6,8]
(18)	Verbal aggression	(1,2)	Increasing	Increasing	Increasing
(19)	Ownership assertion	(1,2)	Decreasing	(6,8)	Increasing

The trends are summarized from Supplementary Figs.  S8 and S9 for initiator’s age and recipient’s age, respectively. For behaviors without a monotonic trend in the probability, we listed the age or age range (in years) of local maximum probability. Closed brackets indicate that the year at the end of an age range is included in the ’peaking age’, whereas open brackets signify that the probability did not reach its maximum in that year.

#### Age

We investigated the effects of age on the predicted probabilities of 19 behaviors, as well as how such effects were moderated by sex, behavioral role (initiator/recipient), and kinship (household status). The predicted probabilities formed three patterns: they either consistently increased with age, consistently decreased with age, or initially increased up to a certain peaking age and then declined. We report the effects of age in the following order of modification factors: household-status, sex, and behavioral role.

First, the effect of age on behavioral probabilities did not show great variations by household status, namely, whether the initiator and recipient were from the same household or not. However, the effect of age was modified by sex for most behaviors, except for *comforting*, *dominating*, and *leading*, which displayed higher probabilities as the initiator’s age increased, regardless of sex. We examined the trends with increasing age and different sexes for the same initiator or recipient, as the age-sex interaction effect did not differ across behavioral roles. For simplicity, we only listed the trends for intra-household interactions, which occurred more frequently than inter-household interactions (Supplementary Fig.  S1b), in Table 3. For inter-household interactions, see Supplementary Table  S8.

Age-sex interaction manifests in multiple ways. First, for certain behaviors, the initiator’s age had distinct and even contrasting effects between girls and boys. When initiators were girls, probabilities of *dirty looks*, *verbal aggression*, *supporting opinions*, and *playful teasing* were predicted to peak before age 5 and then sharply decreased. When initiators were boys, the probability declined consistently for *dirty looks* and increased for the other three behaviors. For recipients of these behaviors, however, the effect of their age did not differ by sex. Across both sexes, older children were more likely to receive dirty looks, face verbal aggression, and have their opinions supported. Children aged 7–10 years were most likely to receive playful teasing. Second, certain behaviors showed age-sex interaction only for recipients, not initiators. For girls, the probability of receiving *physical aggression* peaked at ages 2–3, while that of receiving *aggressive teasing* peaked at ages 4–5. Conversely, the probability for boys to receive *physical aggression* and *aggressive teasing* decreased with age. Girls aged 6–8 years were most likely to experience *ownership assertion* from others, whereas boys became increasingly likely targets of *ownership assertion* with age. 5–6-year-old girls 8–10-year-old boys were most likely to become targets of *taking* behaviors. It’s noteworthy that regardless of sex, toddlers (younger than two) were most likely to initiate physical aggression and assert ownership, and children aged 2–5 were most likely to initiate aggressive teasing and taking resources. Finally, for *sharing* and *tattling*, age-sex interaction existed among both initiators and recipients: girls were most likely to share at 5–6 and tattle at 6–7 years old, but were most likely to receive sharing and tattling when they were 1–3 years old; for boys, the probability of initiating both behaviors peaked before the age of 2 and that of receiving these behaviors increased with age.

Another factor is whether the effect of age on a given behavior differed across initiators and recipients. First, *helping* behavior is the only exception, the probability of which peaked among 3–8-year-old children for both initiators and recipients. Second, the initiator-recipient age difference affected some behaviors. For example, the probabilities of *comforting* and *dominating* were predicted to increase with the initiator’s age but to decrease with the recipient’s age. Besides, older children were less likely to initiate requests for sharing resources, comforting, helping, and access to play, but more likely to receive these requests. Finally, for certain behaviors, initiator’s age and recipient’s age had different but not opposite effects, such as *leading* and *scolding*.

#### Sex

**Figure 3:**
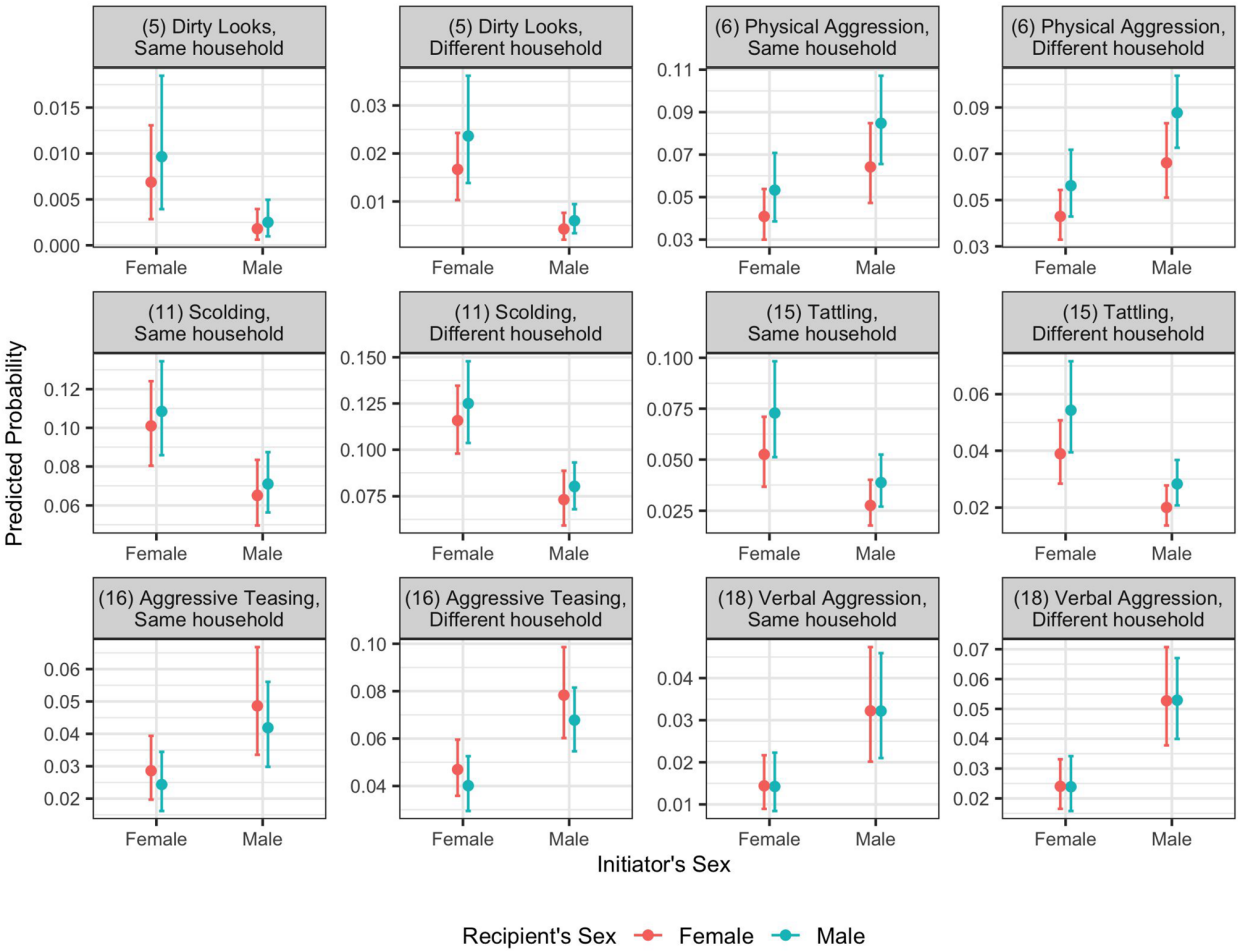
Predicted probabilities of response behaviors as a function of initiator’s sex. Plots are presented for behaviors that are significantly affected by the initiator’s sex. The plots for all behaviors are in Supplementary Fig.  S10. All continuous covariates are held constant at the sample mean. The confidence intervals are the 95% percentile intervals, as calculated from the posterior samples of Model_iF. The coefficients of fixed effects used in the prediction are listed in Supplementary Table  S6.

We predicted the probabilities of 19 behaviors with different sexes of initiators and recipients while keeping their ages at the average of the sample (Fig. 3, Supplementary Figs.  S10, S11). When both the initiator and recipient resided in the same household, a proxy of biological sibling relation, sex did not have a statistically significant impact on the predicted probabilities of any behaviors. However, when the initiator and recipient resided in different households, the sex of the initiator had a significant impact on the predicted probabilities of aggressive behaviors, while the sex of the recipient did not affect any behaviors. Compared to boys, girls were more likely to scold, give dirty looks to, or tattle on others in different households. Boys were more likely to initiate aggressive teasing and verbal aggression toward children from other households. Boys were also more likely to initiate physical aggression, but only towards other boys from other households.

#### Initiator-recipient co-residence

**Figure 4:**
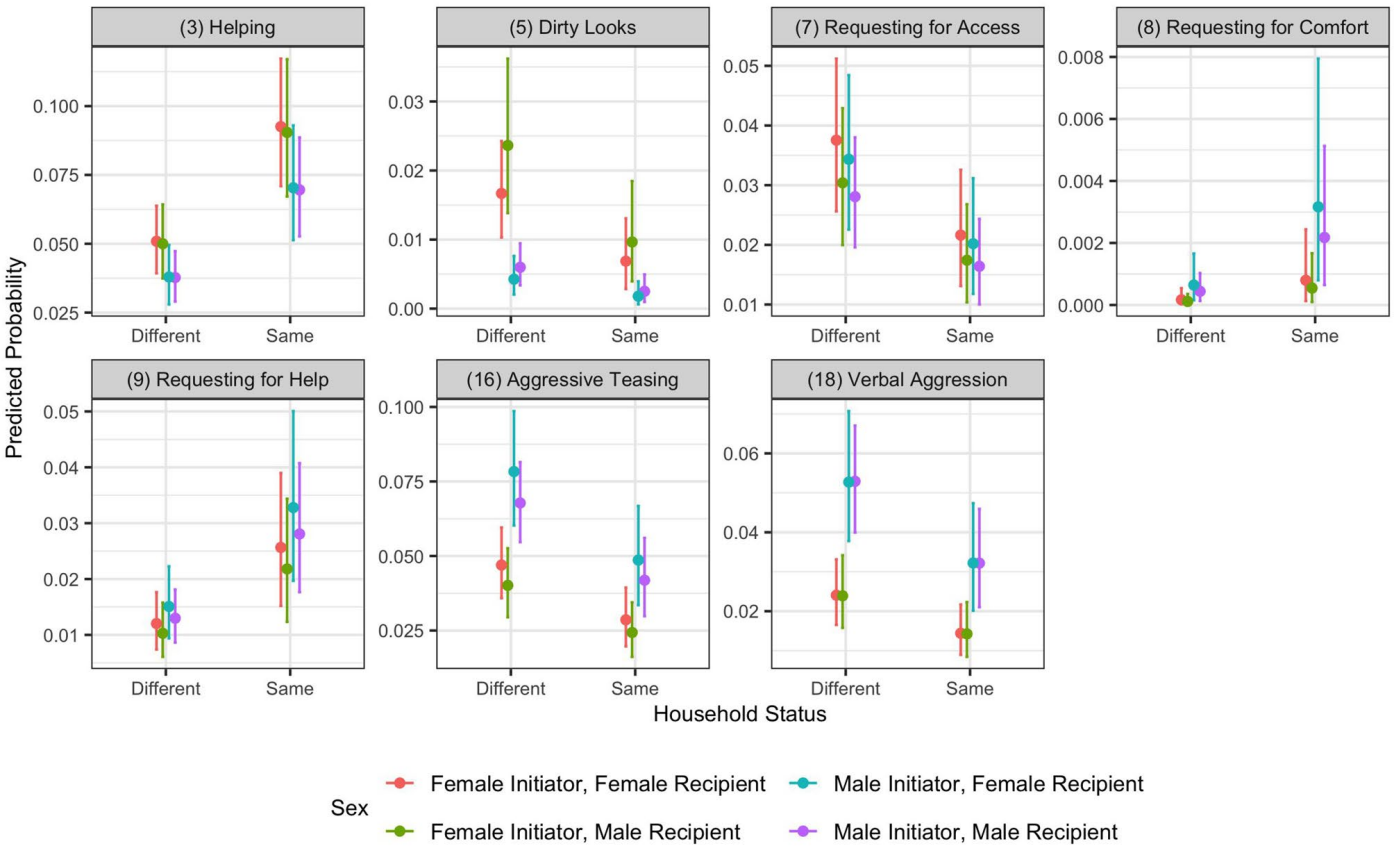
Predicted probabilities of response behaviors as a function of the household status of the initiator and the recipient. Plots are presented for behaviors that are strongly affected by the household status. The plots for all behaviors are in Supplementary Fig.  S12. All continuous covariates are held constant at the sample mean. The confidence intervals are the 95% percentile intervals, as calculated from the posterior samples of Model_iF. The coefficients of fixed effects used in the prediction are listed in Supplementary Table  S6.

We investigated if initiator-recipient co-residence had an impact on the predicted probability of 19 behaviors (Fig. 4), considering four different combinations of sexes, with the ages held constant at the sample mean. Helping behavior was more likely to happen among children from the same household. However, there were no significant differences in the other behaviors.

## Discussion

Our research is a rare study that uses modeling methods to examine naturalistic observations of children’s socio-moral behavior in a historical, non-Western, and rural context. The original dataset, to which we were granted unique access, was the fruit of “the first attempt ever to record in a systematic manner the behavior of Chinese children”^56^, and occupies a significant niche in the intellectual history of anthropology and cross-cultural research on child development. We developed a new coding system from these historical texts and used multilevel multinomial logistic regressions to analyze the effects of demographic factors on a variety of social behaviors. Previous research typically focused on one particular class of behavior (e.g., prosocial or aggression) and analyzed demographic variables such as age and/or sex. Based on the unique data, our study examined multiple prosocial, aggressive, and other locally salient behaviors in an organic community, and added two ecologically valid factors, behavioral role (initiator and recipient), as well as kinship (sibling relation). Our study not only revealed positive correlations within various prosocial behaviors and aggressive behaviors respectively, but also identified linkages between cooperative and non-cooperative behaviors, especially in leadership dynamics. Moreover, we found age, sex, and behavioral roles (initiator/recipient) as key predictors of these behaviors and their effects interacted with each other. Below we discuss the most important findings that generate novel insights on studying culture and moral development in naturalistic settings. We first focus on leadership strategies, as the findings exemplify the value of our approach. We then offer contextualized interpretations of the age-related trends and gendered patterns of other prosocial and aggressive behaviors. Taken together, through re-engaging intellectual history via a new approach, our study challenges classic views on Chinese childhood, illuminate how children’s actual behavior is shaped by but also diverges from cultural ideology, and open up new inquiries for future work.

Our study discovered intricate connections between cooperative and non-cooperative behaviors in the context of leadership dynamics, an important space for moral development and value transmission . Notably, *leading* emerges as the most frequent proactive behavior, highlighting children’s substantial investments in mobilizing and collaborating with their peers to achieve shared objectives. The prevalence of *leading* alongside correlations between distinct behavioral strategies within the same children compels us to closely examine leadership within naturalistic settings. For a given child, *leading*, defined as attempts to persuade another child through non-coercive means, was positively correlated with *dominating*, defined as attempts to coercively impose one’s will on others, and *scolding*, defined as criticizing another child for specific misbehavior. One plausible interpretation is that *leading*, together with *dominating* and *scolding*, may function as complementary strategies to establish and maintain leadership positions during peer interactions. Leaders might intend to use these strategies to directly benefit themselves, but in certain cases, such as in *scolding*, they may also have conferred benefits upon the other party and facilitated the transmission of moral values. *Leading* and *scolding* in peer interactions, although not a prominent topic in previous studies of moral development, were identified from a qualitative, bottom-up perspective as salient ethnographic topics, their significance and interconnectedness further illuminated via statistical modeling. This example shows the novelty and merit of our analytical approach.

Moreover, a closer look at these leadership behaviors revealed possible cultural influences as well as deviation from cultural expectations in real life. For the same initiators, negative correlation between *leading* and *physical aggression* suggests that children in leadership roles refrained from physically bullying others. We also found negative correlations between *scolding* and *physical aggression*, *verbal aggression* and *requesting comfort*. Children who scolded others may strive to become a moral exemplar or authority for their peers and siblings, therefore, were less inclined to engage in physical aggression or display emotional vulnerability due to concerns of shame, a key Chinese moral socialization value^57^. From the perspective of recipients, children targeted by domineering leaders were also the ones more likely to get emotional comfort. Meanwhile the same children were unlikely to receive requests for access to group activities, which means that they did not have leadership over play groups. This suggests that subordinate children did receive care from other children, the dark and the bright side of moral life intertwined. Regarding demographic patterns, all three behaviors, *leading*, *dominating*, and *scolding*, increased with the initiator’s age, which is consistent with local cultural norms that ascribe moral knowledge and authority to older children. We found no significant sex differences in either *leading* or *dominating*, suggesting that girls were as likely to assume leadership roles as boys. Furthermore, girls were more likely to initiate *scolding* than boys, a strategy to establish their authority via moral preaching. Mainstream anthropology scholarship, including the Wolfs’ own works^45,50^, emphasized gender socialization ideology in Chinese culture, i.e., girls submitting to boys, rather than actual experience in childhood, therefore assumed a passive role of girls. Aligned with that gender ideology, the scant observational research of contemporary urban Chinese children found that boys displayed more dominance than girls^48^. This contrast might have resulted from differences in study design, that our data are based on a much more extensive fieldwork with a larger sample size, or that our study employed a more rigorous and sophisticated statistical approach. It might also relate to differences in historical contexts and lifestyles: for example, rural girls in our study had more free-play and and their conduct was less restrained by adult monitoring compared to their urban counterparts; girls in multi-child families had more opportunities and experience in domineering others than urban, singleton children^12^. Regardless, our study offers a precious glimpse into young girls’ agency in leadership dynamics, a topic that has long been obscured in the study of traditional Han Chinese families, and opens up new inquiries about historical continuity and change.

The impacts of age on a variety of behaviors deserve contextualized interpretation, as both the initiator’s and the recipient’s age were important predictors of children’s behaviors. Traditional Chinese societies generally placed more moral demands on older children^45^. Parents from this community expected older children to act as role models for the younger ones and refrain from bullying them^12^, but we found mixed evidence in the actual behavior. Younger children requested prosocial favors from older children, while older children initiated both coercive (dominating) and prosocial behaviors. Older children took the lead in group activities, and offered comfort and assistance, but also asserted their authority by maintaining orders and bullying the children who disobeyed. Younger children tended to look up to older ones for guidance and made requests. They also challenged older ones’ authority through subtle expressions like resentful looks or playful teasing. We found an interesting contrast between *physical aggression*, which peaked among children aged 24–42 months and then declined, similar to what previous studies suggested^36^, and *dominating*, which became more likely as the initiator’s age increased, as shown earlier. As children grew older, they learned to restrict physically attacking others but resorted to other coercive means to impose their own preferences onto others. From a recipient perspective, with increasing age, they solidified their authority and were less likely to be dominated, led, or caught up in a fight. They also sent fewer emotional requests and received less comfort, perhaps the result of learning to control their emotions in accordance with Chinese cultural precepts. Additionally, resource-exchanges during leisure time, such as snacks, rubber bands, tiles, and cards, became petty and trivial for older children, resulting in a decreased likelihood of sharing, taking, and asserting ownership. The finding that older children, although being care-takers and role models, did not actually yield to younger ones in conflict situations, contradicts the important Chinese cultural norm of “older children yielding to younger ones,” a cultural norm that Margery Wolf observed from this community^45^. Hence our study has broader implications for comparing children’s actual behavior with cultural and moral ideologies.

Sex differences in behaviors, although only identified in non-sibling dyads, also shed new light on moral development in Chinese culture. First, we found no sex-differences in prosocial behaviors, which contradicts the female-prosociality bias found in contemporary survey research on Taiwan^58^ but lends support to recent cross-cultural experimental findings^39^. More importantly, patterns in aggressive behaviors offer valuable insights into the relationship between culture, gender socialization and behavior. In non-sibling interactions, boys were more likely to initiate physical aggression compared to girls, but only when the recipients were also boys. Besides physical aggression, we found consistent patterns in other forms of aggression regardless of the recipient’s sex, among non-sibling dyads: boys initiated direct forms of aggression, such as verbally insulting and aggressively teasing others, and such tendencies increased with age; girls displayed subtler forms, including giving dirty looks and tattling, while their verbal aggression and aggressive teasing declined with age. As age increased, boys were less likely to tattle on others, but they were also more likely than girls to become the targets of tattling. Our study therefore reveals a more complex picture of sex differences in aggression than prior observational or self-report studies on Chinese children^48,58^. Girls’ tactics are especially interesting: Tattling can help mitigate conflicts while asserting oneself, through seeking help from external authority. Dirty looks provide a socially acceptable means of expressing discontent without escalating conflicts or drawing potential punishment. These strategies, together with the often ignored girl-to-girl physical aggression, defy stereotypes of docile young girls in traditional Han Chinese families in literary and ethnographic representation, including Margery Wolf’s own works^45,50^. Also, the Wolf’s writings, or previous literature more broadly, have rarely discussed how sex differences in aggression take shape in the specific historical and political contexts. Fine-grained ethnographic analysis of the Wolf Archive, however, shows that fighting and violence among boys might relate to the gangster tradition in the area and the policing culture in Taiwan’s Martial-Law era^12^. These statistical and ethnographic findings, from a typical patriarchal community known for its son-preference and daughter-discrimination, offer insights into both cultural patterning and individual agency and demonstrate historicized and contextualized understanding of gender and aggression .

Kinship, especially sibling relations, is an important factor in children’s social life in this rural community. Although children interacted with their siblings more frequently than with those from other households, this factor alone did not significantly predict their social behaviors. This might be related to children’s residential patterns: instead of segregated apartment buildings housing nuclear families in the cities, this village consists of clustered farmhouses often connected via extensive kinship ties, with ample space for communal life. Even when children were interacting with their siblings, other children of different ages were often present and mingling together in the same group activities, therefore sibling relation became a less important predictor than age. The only exception is *helping*, which more likely occurred between siblings than between non-siblings, probably because siblings looked out for each other when needed^59^. Also, household status did modify the effects of some other factors. Notably, sex differences in children’s behaviors that were significant in non-sibling dyads, e.g. aggression. Patterns of aggression that are aligned with well-established findings in previous works, i.e., male-bias in physical aggression, disappeared when the initiator and the recipient were from the same household. The ethnographic analysis did show that sibling fighting at home was a salient theme in this community^12^, which can partly explain why sibling relation overrides sex differences in predicting aggressive behaviors. Future work should pay more attention to sibling dynamics when examining gendered moral development.

The present study also has several limitations. First, the effects of kinship on children’s behavior remains an intriguing question that merits further analysis beyond the scope of this study. The current study used household number as a generic index for kinship, especially sibling relationships, without specifying birth order, family size, or the nature of family ties, e.g., biological or adoptive siblings. Because most families in this sample had more than two children, and some still adopted children–a local custom that declined during the Japanese-rule but was not eliminated by the 1960s^56^-future work can disentangle how these different factors impacted children’s behavior. Also, although our data came from 70 distinct households, many households were connected via kinship. A majority of the villagers descended from the same Chinese immigrants in southern Fujian who had arrived in the region during the eighteenth and nineteenth centuries. A large joint family lived in this village, as well as some nuclear or stem family households, as part of the Chinese family cycle^44^: For example, when two brothers get married, one large household may split into two smaller ones for their respective families. As a result, we may apply the ideas and techniques of Gaussian process regression to the multinomial model. Instead of considering discrete boundaries between households, we may employ a matrix of distances between pairs of observed behaviors. However, anthropologists may find such statistical models unnecessarily complex, and it remains an open question if this dataset from only one village is suitable for such increased statistical complexity in analysis.

Given its historical, naturalistic datasets, the Wolf Archive also affords researchers the opportunity for further reflections and analyses. First, our model only considered a limited number of individual-level demographic predictors, but other household-level variables may matter too. Household SES status might be a predictor of children’s behaviors if we combine the current behavioral data with SES data inferred from other fieldnotes left behind by Arthur Wolf. Moreover, as natural observations also include reactive behaviors, we may extend our analysis to reactive behaviors. Since some proactive and reactive behaviors are matched (for example, *dominating* vs. *submitting*), we can explore the mechanisms and demographic patterns of behavioral contingencies, reciprocity, and social relations, via social network analyses. Also, besides dyadic interactions, quantifying and modeling multi-agent dynamics in the raw data is a promising next step. Moreover, given the importance of age in predicting children’s behaviors in this dataset, further analyses should closely examine developmental patterns of particular behaviors broken down to different age groups, from infancy to early adolescence, or model the longitudinal trajectories of behaviors across the two-year research time span, and interpret those behavioral patterns in the local context. Additionally, given the complexity of naturalistic observations, we can eventually integrate quantitative and ethnographic, qualitative approaches, to better understand human behavior in its socio-cultural contexts^12^. Finally, this dataset only captures children’s social life in one particular time-space, and future work should gather more diverse datasets of naturalistic observations from old and new ethnographic records to ensure enriched cross-cultural comparison and generalization.

## Methods

### Original data

The study is a secondary analysis of historical texts, field-notes collected by the late anthropologist Arthur Wolf in a Hokkien-speaking village in rural Taiwan, 1958–1960, as part of his dissertation research. His research was approved by Cornell University and conducted according to the relevant guidelines and regulations at that time in the U.S. Children’s social behavior was observed inside the village, at home, and at the elementary school outside the village. The first-hand witness of children’s social behavior was Arthur Wolf’s research assistant, an Taiwanese teenager girl who spoke the local language and was trusted by local children and their families because of her personality. She observed children’s naturalistic behaviors in meticulous detail documented them in systematic episodes, written in Chinese. On the same day, the research assistant then reported her observations to Margery Wolf, who was the anthropologist Arthur Wolf’s wife and performed the role of a “scribe” at that time^60^. Margery Wolf then translated these observations into English and typed them up. These typewritten notes, preserved in Arthur Wolf’s private library in Northern California, constitute the original data that our study is based on. All the observations were indexed by their event information (data, time, location) and by the ID of the participants, both initiator and recipient. Demographic information was also collected in a systematic manner, such as the age at observation, sex, and household number of all the people involved.

The data collection approach, the excellent local research assistant, and the prolonged fieldwork made the observational texts in the Wolf archive even richer and rarer, compared to the SCS materials. According to the SCS field guide^18^, Child Observation should focus on a predefined set of social situations. Wolf’s RA, in contrast, reported everything she saw the children doing and saying and how other people were involved or reacted, all in spontaneous episodes rather than waiting for a particular situation to occur. Also, while the SCS field guide designed CO as “short excerpts of behavior rather than extended interaction sequences,” Wolf’s RAs did much better than that, by violating the instructions and recording extended behavioral sequences faithfully^56^. Therefore, although Wolf intended to follow the SCS design and target children ages 3–12 (calculated at the beginning of study), it turned out that these observational records contained abundant information about a much larger sample of children which also includes those who were younger than 3 or older than 12.

### Digitization and secondary analysis

Arthur Wolf’s original fieldnotes were housed in his residence. With unique permission to access and use them, we digitized these materials into machine-readable files. We obtained ethical approval from the Internal Review Board of the University of Washington for analyzing these fieldnotes. We did secondary analysis on de-identified data, including naturalistic observation texts and demographic and household information. In both types of texts all the participants were labeled by numbers. All analyses were carried out in accordance with relevant guidelines and regulations.

We assigned a unique ID to each of the 1677 observational episodes, and manually coded all the episodes according to a standardized behavioral coding protocol we designed. We designed this new behavior-coding protocol that includes about fifty social interaction themes. In this study, we focused on child-to child dyadic interactions, excluding child-adult interactions such as command-obey, as well as all behaviors that were not directed from an initiator to a recipient, such as agricultural work and housework. We also limited the target population to children younger than 13 years old at the end of the two-year fieldwork. Though there were 1677 observational episodes, we amalgamated responses over all observation episodes. The final dataset analyzed in this paper contains 19 categories of proactive, dyadic directional behaviors (Supplementary Table  S1), adding up to 6507 entries of behaviors over two years, and 18 categories of reactive 1-to-1 directional behaviors, adding up to 2344 entries. Since the proactive (e.g., dominating) and reactive behaviors (e.g., submitting) were coded based on different schemes, for clarity of analysis and convenience of interpretation in this study we focus on just the proactive behaviors. Note that *sibling care*, a proactive behavior, was excluded from the study because it can only be exhibited by children who have younger siblings, whereas other behaviors do not have this constraint. The effect of attending to siblings or not will conflate risk factors for those who could care but did not versus those who did not care because they had no siblings.

### A sample episode

Here is a sample observational episode in this dataset: Observation ID: 28, Date: 08/03/1959, Location: Two logs near the big tree. Observer: MC. Observation content: 493, 157, 145, and 144 were sitting on the two logs. The others were nearby. MC asked them if they had eaten. 145: Let’s not answer her. No one answers her and they laugh. MC did not pay any attention. 144 answered her. He said: I ate two halves. (He meant to say that he had eaten, but mispronounced the words. Actually, he hadn’t eaten.) 157: Yeh, you’ve already eaten two halves of fruit. How big your stomach is? Oh! You’ve eaten two halves. You must be very full, etc. 157 kept yelling these comments over and over and everyone was laughing at 144’s mistake. 144 finally got angry and hit 157 lightly, saying: Quit it! Quit it! The children continued to laugh and 157 kept saying this. 144 started to tickle 157. They laugh. 131 came to tickle 157 also. 157 stood up and tried to catch 131, but he missed him. 157–144: Stand up and let’s fight. We’ll see who wins. 144 stands up smiling and they wrestle. The other children are still laughing because 157 continues to tease 144 about his big stomach, etc. as they wrestle. 144 is beginning to get angry and 157 sees this. 157 runs away from 144 and yells: Oh, your stomach is so big from eating two halves. 144–157: I’m going to knock you down on the ground. 157 runs away, sits down again. 157 comes near and 144 stands up. Then 157 runs away. This is repeated several times with 144 threatening to hit 157 and 157 teasing 144 about the two halves he has eaten. 145–157: I’m going to hit you, too. Quit it! Finally, 144 ignores him. 157 came and sat in front of 144 and started to sing a song. He changed the words to call 144 a “Big Forehead”. (144’s forehead protrudes a little) and soon all the children were singing this. 144–157: I’m going to hit you. He picks up his slingshot and says: I’m going to hit you with my slingshot. 157 continues to sing. 144 hit him with the slingshot, but 157 kept singing. 144 turned to 493: Why are you laughing at me? 493: Copulate with your mother. I’ll1 not laughing at you. I’m just singing a song. 144 and 493 swear at each other and punch each other with their shoulders (they are sitting next to each other). 157 is still singing. 144 is very angry now. 144: I’m going to hit you. 157 runs home with 144 after him. 157 goes into House 14. 144 sits in front and says he’s going to wait until 157 comes out. 157 says something to 144. 144 grabs a stick and runs into the house and hits him. 140 is in the kitchen and says: Are you two still fighting in there? P comes out and 157 comes out too. 157 begin to tease him again. 144 angrily chases him again. 157 hides behind a tree and sings the song. 144 still has the stick in his hands. 145 turns to 131: You No Teeth (131 is missing some teeth). 131–145: You Eleven Fingers (145 has 11 fingers). 131 hit 145 on the face. 145 ran to 144. 144–131: What are you laughing at? 144 threw a rock at 131 but misses. 131 keeps singing the song.144 starts to chase him but 131 runs home. Then 144 turned and chased 157 again. 157 continues to sing. 153 came out. 144–153: 157 is scolding me. 157: I’m not. I’m not. I’m just singing a song. He continues to sing. 153–144: What did he scold you about? 144: He calls me Big Forehead. 153–157: Why are you scolding anyone? Go find the ducks. 157 went to find the ducks and 144 went home. 149 called from the house: Quit fighting, 157, and go the ducks. They aren’t in the house now. 157 ignored her.

### Statistical approach

To analyze the behavior data we fitted multilevel multinomial logistic regressions following Koster and McElreath^54^. This approach accounts for the multinomial character of the response variable while also accounting for children’s repeated observations across observation episodes. We denote the behavioral themes as $1,2,\ldots ,19$, with the probability of observing each behavior between initiator *i* and recipient *j* being $\pi _{1,i,j},\ldots ,\pi _{19,i,j}$.

Children can exhibit a set of behaviors due to various unobserved factors, resulting in the clustering, i.e. dependence, of the behavioral variables by individual. Behaviors may also cluster at the household level in the sense that the members of the same household tend to exhibit similar behaviors. Thus, we used multilevel modeling to account for this potential higher-level clustering. Our statistical model allowed the probabilities of exhibiting each behavior to vary across initiators, recipients, and households, even with the same age and sex, by adding random effects. Our model can be summarized by 18 equations that contrast the odds of exhibiting all the different behaviors instead of a reference behavior, behavioral theme 19. Multilevel multinomial logistic behavioral models that apply generalized linear mixed model principles are a good fit for the structure of observational data obtained using scan sampling techniques. Time-varying covariates could be included in these models (e.g., age), and the dependence between measurements made on the same child and the imbalance in the population sample was addressed by the addition of random factors. More notably, by displaying correlated random effects across the response categories, the models provided insights into the behavioral patterns. It is important to highlight that when there are few occurrences of a certain behavior, the posterior distribution can simply reflect the prior of the model for that rare behavior. In our study, though behavioral themes *(1) Comforting*, *(5) Dirty Looks*, and *(8) Requesting for Comfort* had relatively small numbers of occurrences, we did not combine these behaviors from the original coding scheme because we believed that these behaviors are salient in understanding children’s social life and also failed to come up with a reasonable scheme to combine them. Despite the relatively small numbers of these three behaviors, we expected that there would not be major problems fitting the model. In fact, the prior and posterior distribution of the parameters differed for these behaviors (Supplementary Figs.  S10,  S11).

We fitted four models named by the random and fixed effects included in each of them. Model_i and Model_iF include individual random effects from the initiator and recipient, while Model_ih and Model_ihF include random effects from both the individual and household of the initiator and recipient. Model_i and Model_ih did not include any fixed effect from predictor variables, whereas Model_iF and Model_ihF included predictor variables.

#### Model_i—individual-level random effects only.

The probabilities of all the distinct behaviors sum to one, so we have

$$\begin{aligned} \sum _{k=1}^{19}\pi _{k,i,j}=1 \text { for each initiator }i\text { and recipient } j. \end{aligned}$$

For each observed behavior, the log-ratio comparing the odds of initiator *i* and recipient *j* exhibiting each of the 18 pivot behaviors instead of the reference behavior is assumed to be given by

$$\begin{aligned} \text {log}\left( \frac{\pi _{k,i,j}}{\pi _{19,i,j}}\right) =\alpha _k+\nu ^I_{k,i}+\nu ^R_{k,j};~k\in \{1,\ldots ,18\}, \end{aligned}$$

where each $\alpha _k$ is an intercept that contrasts the behavior *k* against the reference category and where $\nu ^I_{k,i}$ and $\nu ^R_{k,j}$ are person-specific effects for initiation and reception of behavior theme *k* in initiator *i* and recipient *j*, respectively. Across all the behavioral themes we assume the priors

$$\begin{aligned} \left( \nu ^I_{1,i},\cdots ,\nu ^I_{18,i}\right) \overset{i.i.d}{\sim } \text {Normal}(0,\Sigma _{\nu \_I}),~\Sigma _{\nu \_I}= \begin{bmatrix} \sigma ^2_{\nu ^I_{1}} &{} \cdots &{} \sigma _{\nu ^I_{1,18}}\\ &{} \ddots &{} \vdots \\ &{} &{} \sigma ^2_{\nu ^I_{18}} \end{bmatrix}, \text{ for } i=1,\ldots , 213, \\ \left( \nu ^R_{1,j},\cdots ,\nu ^R_{18,j}\right) \overset{i.i.d}{\sim } \text {Normal}(0,\Sigma _{\nu \_R}),~\Sigma _{\nu \_R}=\begin{bmatrix} \sigma ^2_{\nu ^R_{1}} &{} \cdots &{} \sigma _{\nu ^R_{1,18}}\\ &{} \ddots &{} \vdots \\ &{} &{} \sigma ^2_{\nu ^R_{18}} \end{bmatrix}, \text{ for } j=1,\ldots , 213. \end{aligned}$$

These state that person-specific intercepts, $\nu ^I_{1,i},\ldots ,\nu ^I_{18,i}$ and $\nu ^R_{1,j},\ldots ,\nu ^R_{18,j}$, both follow a multivariate normal distribution with mean zero and their own positive-definite $18\times 18$ variance-covariance matrix, across behaviors other than the reference category. The off-diagonal elements of these matrices represent the individual-level pairwise covariance between random effects among behaviors 1–18, while the diagonal elements signify the individual-level variance of random effects within each behavior.

The intercept $\alpha _k$ for a behavior *k* represents the log-odds of exhibiting that behavior relative to the reference behavior, assuming that all random effect terms are zero. In other words, $\alpha _k$ is the mean log-odds over all subjects for exhibiting behavior *k* compared to the reference behavior. Random effects were introduced to account for varying occurrence probability of behavior *k* rather than the reference by different initiators and recipients. A positive individual-level random effect for the initiator *i*, $\nu ^I_{k,i}>0$, implies that initiator *i* is more likely to exhibit behavior *k* instead of reference behavior than the population average, and vice versa. Instead of the magnitude of random effect from each individual, we are interested in the variance of the individual random effects in each behavioral category, providing insights into the extent to which unobserved individual-level factors contribute to the observed variation in the occurrence of each behavior, compared to other sources of variation. A large variance of the individual-level random effects for the initiator for behavior *k* implies that the probability of exhibiting behavior *k* instead of reference behavior varies greatly among the initiators. We can also obtain the pairwise correlations across all behaviors (except for the reference) from the corresponding pairwise covariances (e.g., behavior *k* vs behavior *l* for initiators, $\rho ^I_{k,l}=\sigma _{\nu ^I_{k,l}}/(\sigma _{\nu ^I_{k}}\sigma _{\nu ^I_{l}})$), which provided insights into the co-occurrence of different behaviors by initiators and recipients. When the initiator’s individual-level random effect has a positive correlation between two behaviors *k* and *l*, $\rho ^I_{k,l}>0$, an individual who is more (or less) likely to initiate behavior *k* is also more (or less) likely to initiate behavior *l*. Conversely, a negative correlation implies that initiators who are more (or less) likely to exhibit behavior *k* are less (or more) likely to exhibit behavior *l*. The interpretation of individual-level random effects and pairwise correlations for the recipient is similar. We chose behavioral theme 19, *ownership assertion*, as the reference behavior because correlations of random effects between *ownership assertion* and other behaviors were of less interest than correlations of random effects between the remaining peer-interactive behaviors.

Because of the considerable complexity of this model, we fit it using Bayesian methods, enabling the use of highly-flexible Markov Chain Monte Carlo (MCMC) algorithms. Specifically, we assigned standard normal priors to the intercepts, $\alpha _k$. In theory, the covariance structure of the individual-level random effects, $\Sigma _{\nu \_I}$ or $\Sigma _{\nu \_R}$, can be decomposed into a correlation matrix and a vector of element-specific variances, or “scale” terms^61^. To improve the computational efficiency and arithmetic stability of MCMC simulations, we employed a non-centered parameterization of the random effects based on a Cholesky factorization of the correlation matrix. This decomposition represents the Hermitian positive-definite correlation matrix as a product of a lower triangular matrix and its conjugate transpose^62,63^. We set a Cholesky factorized prior with shape equal to 2 for the parameterized correlation matrix.

#### Model_iF—fixed effects from individual characteristics

To investigate if some demographic characteristics of children were highly influential factors in children’s behaviors, we included some predictor variables as fixed effects in our model. By interpreting the coefficient of the fixed effects in our models, we are able to improve our understanding of the various factors influencing children’s development and behaviors.

In addition to the individual-level random effects presented in Model_i, Model_iF includes individual-level variables for age and sex, as well as their interaction. To investigate how children from the same or different families influence the interaction between them, Model_iF also incorporates an indicator of whether the initiator and recipient were from the same household. We standardized the continuous predictors^64^, i.e., ages of initiators and recipients, to make sampling (from the posterior distribution as described later) more efficient. Specifically, the variables were shifted and rescaled to have a mean of zero and a standard deviation of one. Model_iF had the form

$$\begin{aligned} \text {log}\left( \frac{\pi _{k,i,j}}{\pi _{19,i,j}}\right) =\alpha _k+\nu ^I_{k,i}+\nu ^R_{k,j} +\sum _m\beta _{k,Im}x_{Im}+\sum _m\beta _{k,Rm}x_{Rm}+\beta _{k,H}x_{H};~k\in \{1,\cdots ,18\}, \end{aligned}$$

where $x_{Im}$ and $x_{Rm}$ are the fixed effect *m* from the initiators and the recipients, respectively, and $x_{H}$ is a binary indicator variable that reflects whether the initiator and recipient in each behavior observation belonged to the same household or not. The individual-level effects $\nu ^I_{k,i}$ and $\nu ^R_{k,j}$ are assumed to follow the same multivariate normal distribution as in Model_i, and $\pi _{k,i,j}$ always sum up to 1 for all *i* and *j*. Model_iF summed over all fixed effects included in the model.

In this mixed effects model, the intercept $\alpha _k$ represents the log-odds of exhibiting behavior *k* relative to the reference behavior, when all predictors and random effects are zero. Specifically, it represents the average log-odds of behavior *k* that occurs between female initiators and recipients, who are of the same age as the sample average, and come from different households. The coefficient of a predictor, denoted as $\beta _{k,Im}$, $\beta _{k,Rm}$, or $\beta _{k,H}$, measures the effect of a one-unit increase in that predictor, $x_{Im}$, $x_{Rm}$, or $x_{H}$, on the average log-odds of exhibiting behavior *k* instead of the reference behavior. In other words, it tells us how much the average log-odds of behavior *k* change when we increase the corresponding predictor by one unit, holding all other predictors constant.

The prior distributions on the parameters in this model are the same as those in Model_i, with additional standard normal priors assigned to the independent fixed effects parameters $\beta _{k,Im}$, $\beta _{k,Rm}$, and $\beta _{k,H}$.

#### Model_ih—both individual and household-level random effects

In addition to the individual-level random effects presented in Model_i, Model_ih included random effects from both the initiator’s household [*i*] and the recipient’s household [*j*]. Model_ih had the form

$$\begin{aligned} \text {log}\left( \frac{\pi _{k,i,j}}{\pi _{19,i,j}}\right) =\alpha _k+\nu ^I_{k,i} +\nu ^R_{k,j}+h^I_{k,[i]}+h^R_{k,[j]};~k\in \{1,\cdots ,18\}, \\ \left( h^I_{1,[i]},\cdots ,h^I_{18,[i]}\right) \overset{i.i.d.}{\sim }\ \text {Normal}(0,\Sigma _{h\_I}),~\Sigma _{h\_I}= \begin{bmatrix} \sigma ^2_{h^I_{1}} &{} \cdots &{} \sigma _{h^I_{1,18}}\\ &{} \ddots &{} \vdots \\ &{} &{} \sigma ^2_{h^I_{18}} \end{bmatrix}, \text{ for } [i] = 1,\ldots , 70, \\ \left( h^R_{1,[j]},\cdots ,h^R_{18,[j]}\right) \overset{i.i.d.}{\sim }\ \text {Normal}(0,\Sigma _{h\_R}),~\Sigma _{h\_R}= \begin{bmatrix} \sigma ^2_{h^R_{1}} &{} \cdots &{} \sigma _{h^R_{1,18}}\\ &{} \ddots &{} \vdots \\ &{} &{} \sigma ^2_{h^R_{18}} \end{bmatrix}, \text{ for } [j] = 1,\ldots , 70, \end{aligned}$$

where $h^I_{k,[i]}$ and $h^R_{k,[j]}$ are household-level random effects for initiator *i* and recipient *j*, respectively. The individual-level effects $\nu ^I_{k,i}$ and $\nu ^R_{k,j}$ follow the same multivariate normal distribution as in Model_i, and all $\pi _k$ always sum up to 1. This extended model has nested random effects since each individual is uniquely associated with only one household. Since the personal random effects should be nested within households, the random terms, while not identical, are more likely to be similar within a household versus between households.

Including the household-level random effects changes the interpretation of the individual-level random effects. The individual-level random effects are interpreted as the deviation from the household-level average rather than the population average. A positive individual-level random effect for the initiator *i*, $\nu ^I_{k,i}>0$, now implies that the probability of exhibiting behavior *k* instead of the reference behavior is higher for initiator *i* than the average likelihood within the corresponding household, and vice versa. This means that the influence of unobserved individual-level factors on the likelihood of exhibiting a particular behavior is being measured relative to the average behavior of the household rather than the population as a whole. Furthermore, when a household-level random effect of the initiator is positive, $h^I_{k,[i]}>0$, individuals in that household [*i*] has an above-average chance of initiating behaviors *k* instead of the reference behavior, and vice versa. The interpretations are similar for recipients.

The prior distributions for the intercepts and the individual-level random effects in this model are the same as those in Model_i, with additional Cholesky factorized priors with a shape equal to 2 assigned to the parameterized correlation matrices of household-level random effects, $\Sigma _{h\_I}$ and $\Sigma _{h\_R}$.

#### Model_ihF—fixed effects from individual characteristics

In addition to the individual-level random effects presented in Model_iF, Model_ihF included random effects from both initiator’s and recipient’s household. Model_ihF had the form

$$\begin{aligned} \text {log}\left( \frac{\pi _{k,i,j}}{\pi _{19,i,j}}\right) =\alpha _k+\nu ^I_{k,i} +\nu ^R_{k,j}+h^I_{k,[i]}+h^R_{k,[j]}+\sum _m\beta _{k,Im}x_{Im} +\sum _m\beta _{k,Rm}x_{Rm}+\beta _{k,H}x_{H};~k\in \{1,\cdots ,18\}. \end{aligned}$$

The prior distributions for the intercepts and independent fixed effects in this model are the same as those used in Model_iF. Similarly, the prior distributions for the covariance structures of random effects are the same as those employed in Model_ih.

### Estimation of parameters

Since this multilevel multinomial logistic model is not implemented in standard software, we fitted a Bayesian version of the model using Markov Chain Monte Carlo (MCMC) estimation rather than the commonly used maximum likelihood method. In particular, the inference is based on the expectations of posterior quantities, such as posterior means and standard deviations of parameters.

We used R’s *RStan* package^61^ to facilitate MCMC. *RStan* is the R interface to *Stan*, which is a state-of-the-art platform for statistical modeling and high-performance statistical computation. Users can specify log density functions in Stan’s probabilistic programming language and get full Bayesian statistical inference via Hamiltonian Monte Carlo sampling (HMC), which is a family of MCMC algorithms^65^. Stan is preferred over the older but widely used BUGS software due to its considerably higher efficiency and faster running speed^66^. We employed weakly informative priors for the parameters of fixed effects and variance-covariance matrices of random effects as described beside the statement of models. We performed prior sensitivity analysis and validated the use of the weakly informative priors. We ran each model on three chains, each with 10000 iterations and a warmup of 5000 iterations. We confirmed model convergence by examining the trajectory plot of the chains and the R-hat Gelman and Rubin convergence diagnostic.

### Analysis of raw output

We utilized the *rethinking* package to prepare data, summarize the posterior, and plot model predictions^67^. We compared the predictive performances of the four models based on the Widely Applicable Information Criterion (WAIC)^55^. We estimated the Cholesky matrix using HMC chains and computed the correlations between the random effects across behaviors via recomposition from the lower triangular matrix and its conjugate transpose. This allowed us to determine if individuals who engage in more of the first behavior also tend to engage in more or less of the second behavior (relative to the reference category).

The coefficients of the fixed effects in Model_iF and Model_ihF are interpreted as the effect of a one-unit difference in one predictor on the log-odds of exhibiting behaviors 1 to 18 instead of the reference behavior after adjusting for other predictors. However, the interpretation of the coefficients is rather awkward. It would be much easier and straightforward to interpret the effects of a predictor on each behavior, rather than on a contrast between two behaviors. Besides, Retherford and Choe^68^ noted that coefficients (or odds ratios) are not only difficult to interpret but may even be misleading because the sign of a coefficient may not reflect the direction of the effect of the predictor on either of the response probabilities being compared (i.e., $\pi _k$ and $\pi _{19}$). Thus, we calculated the predicted response probabilities for each of the 19 behaviors from the estimated coefficients of the fixed effects using a random effect value of zero, giving the predicted probabilities of behavior between an “average” recipient and an “average” initiator. We plotted the predicted probabilities with one predictor varying at a time while holding other predictors constant, together with 95% credible intervals incorporating uncertainty in the fixed effect parameters.

### Supplementary Information

Supplementary Information.

## Data Availability

The raw fieldnotes are part of a private historical archive not available to the public yet. All de-identified and processed data and associated R scripts are available at https://github.com/zhiningsui/children_behavior_multinomial_analysis.
