# Supplementary material for: Modeling children’s moral development in postwar Taiwan through naturalistic observations preserved in historical texts
Source: Sci Rep. 2024 Apr 21;14:9140. doi: 10.1038/s41598-024-59985-6 (PMC11033267; doi:10.1038/s41598-024-59985-6)
Supplement: Supplementary file 1 — Supplementary Information. [file 41598_2024_59985_MOESM1_ESM.pdf]

Supplementary Information for: Modeling children's moral development in postwar Taiwan through naturalistic observations preserved in historical texts

Zhining Sui[1, 2], Qinyan Wang[3, 4], and Jing Xu[5,*]

[1]Department of Biostatistics, University of Washington, 1410 NE Campus Parkway Seattle, WA 98195, USA.
[2]Department of Biostatistics and Computational Biology, University of Rochester, 265 Crittenden Boulevard, Rochester, NY 14642, USA.
[3]Department of Linguistics, University of Washington, 1410 NE Campus Parkway Seattle, WA 98195, USA.
[4]Amazon.com, Inc. 400 9th Ave N, Seattle, WA 98109
[5]Department of Anthropology & eScience Institute, University of Washington, 1410 NE Campus Parkway Seattle, WA 98195, USA.
[*]email: jingxu1983@gmail.com

Correlations between different behaviors for the same initiators or recipients: Model_iF

Since the random effects are estimated relative to the prediction obtained by the predictor variables in the model, when we account for the additional predictors in Model_iF, the random effect may change its magnitude and sign. Our results show that most of the correlations of individual-level random effects were weaker in models with additional fixed effects from predictors (Supplementary Fig. S7). More importantly, there was no significant correlation of individual random effects across all behaviors for both initiators and recipients (Supplementary Table S3). The observed changes in significant correlations are associated with how the estimated random effects are affected by the fixed effects.

Interpretation of models with additional household-level random effects: Model_ih and Model_ihF

The Model_ih and Model_ihF incorporated additional random effects for households based on Model_i and Model_iF, respectively. However, as suggested by the WAIC comparison, the inclusion of household-level random effects did not greatly improve the predictive performance of the models. This is because the fixed effects that exhibit significant effects are not defined at the household levels in the data structure. Supplementary Table S7 shows that the estimated coefficients of fixed effects did not change substantially when incorporating additional household random effects compared to Model_iF. The predicted probabilities of behaviors obtained from Model_ihF closely corresponded to the those obtained from Model_iF (Supplementary Fig. S15,S16,S17,S18,S19).

These extended models also estimated the variances and correlation matrices for the individual-level random effects, but the interpretation has changed with the inclusion of household-level random effects. The individual-level effects now represent deviations from the household-level average. The variance estimates of the individual-level effects capture the within-household variation among individuals, rather than the variation across individuals in the overall study population (Supplementary Table S9). Additionally, with a data structure that consists of 213 children among 70 households, the estimation of household-level random effects had limited precision. Thus, there is limited inferential insight to be gained from examining the variance estimates of their correlations (Supplementary Table S4, S5).

Supplementary Figure

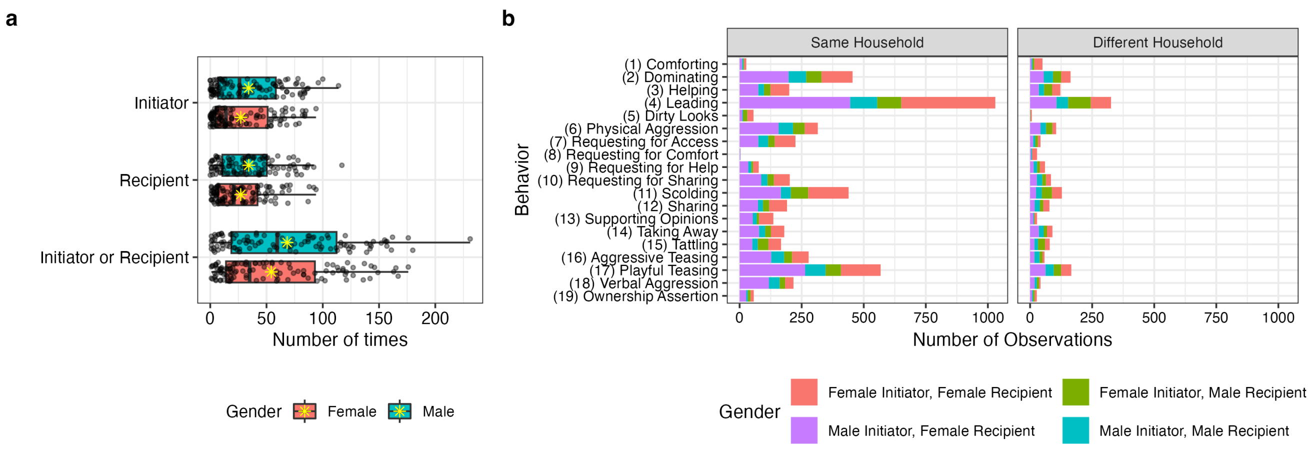

Supplementary Figure S1. Descriptive analysis of relationship between behavioral role, sex, and household status. (**a**) Number of times a child being an initiator, a recipient, or either, by different sexes. The yellow asterisks demonstrate the mean number of times. (**b**) Number of observed behaviors by sex and household status.

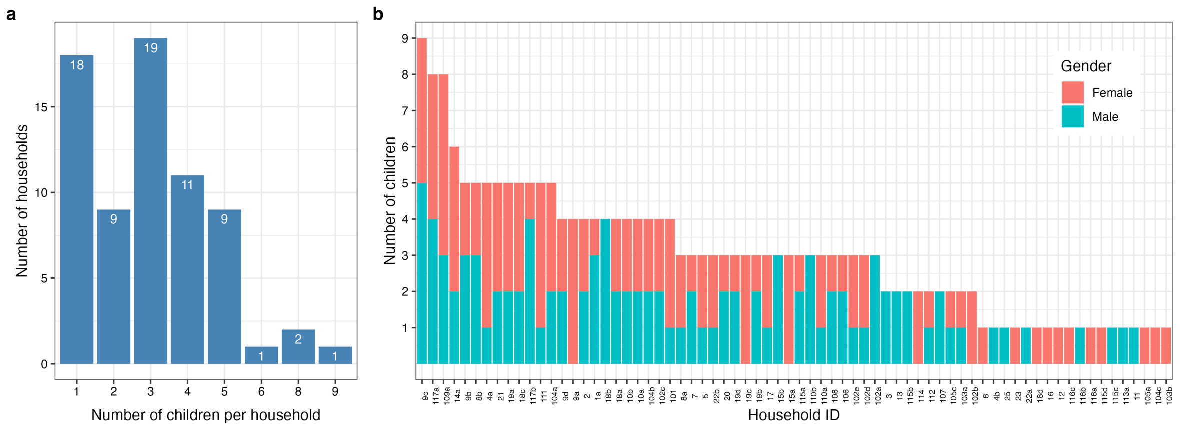

Supplementary Figure S2. Descriptive Statistics for the household. (**a**) Distribution of children per household. (**b**) the sex composition of each household.

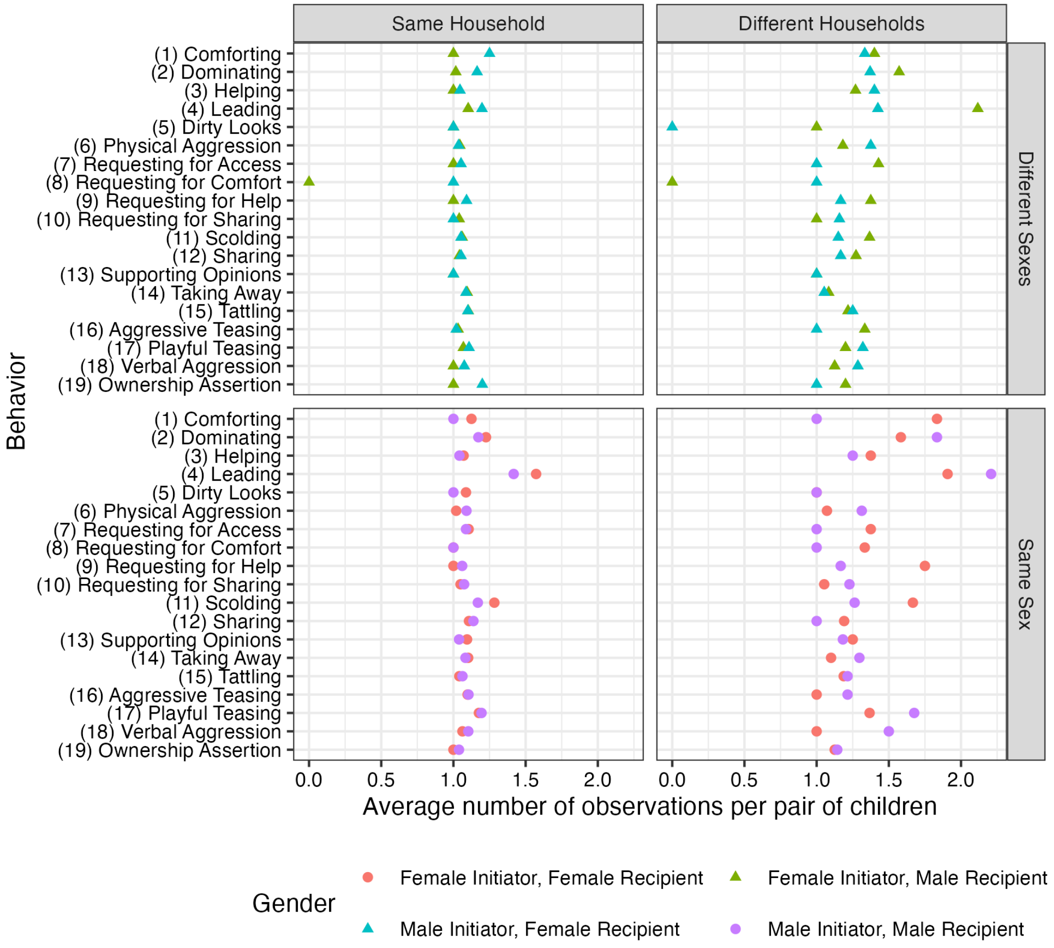

Supplementary Figure S3. Average number of observations per pair of children, whether from the same or different households, and of the same or different sexes.

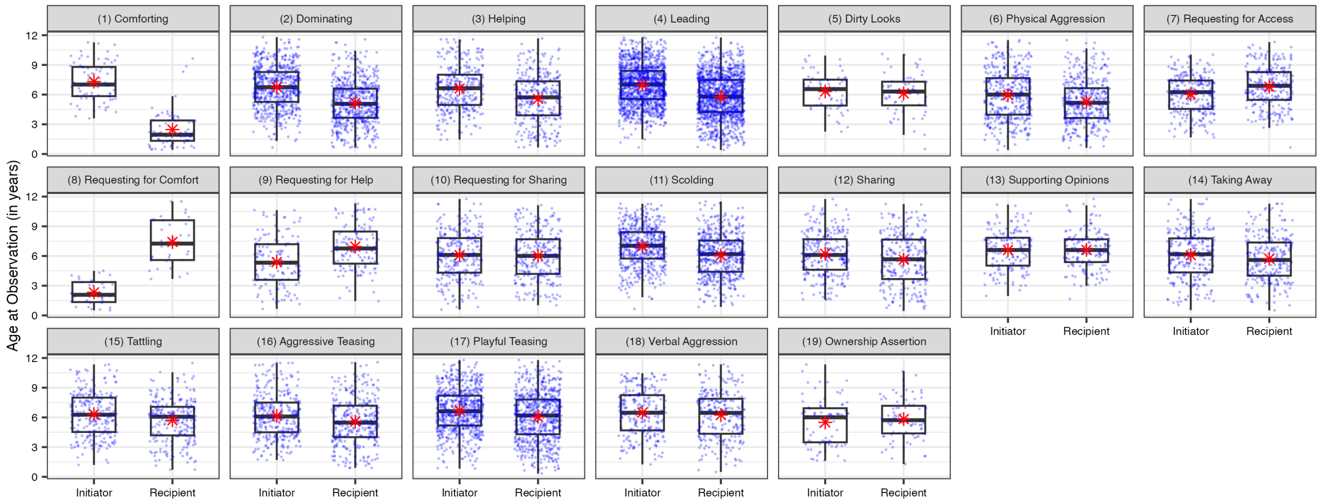

Supplementary Figure S4. Age of initiator and recipient at each observation of each behavior. The red asterisks demonstrate the mean age at observation.

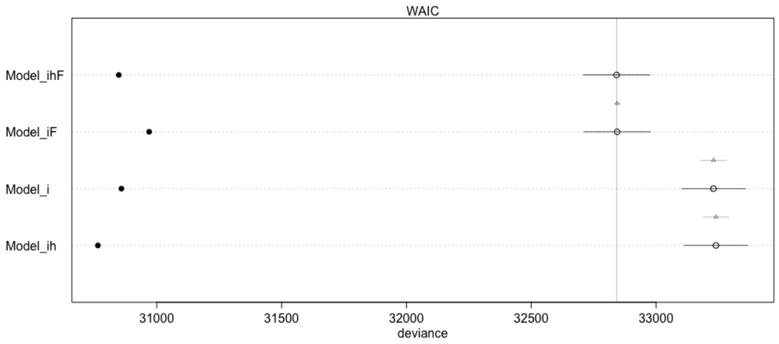

Supplementary Figure S5. Visualization of WAIC values from Table S1. The open points are the WAIC for each model. Dark lines through these points represent the standard deviation of the WAIC. The gray triangles above each WAIC are the difference between that model and top-ranking model, with the standard deviation of that difference depicted by gray segments. The filled points are the in-sample deviance of each model.

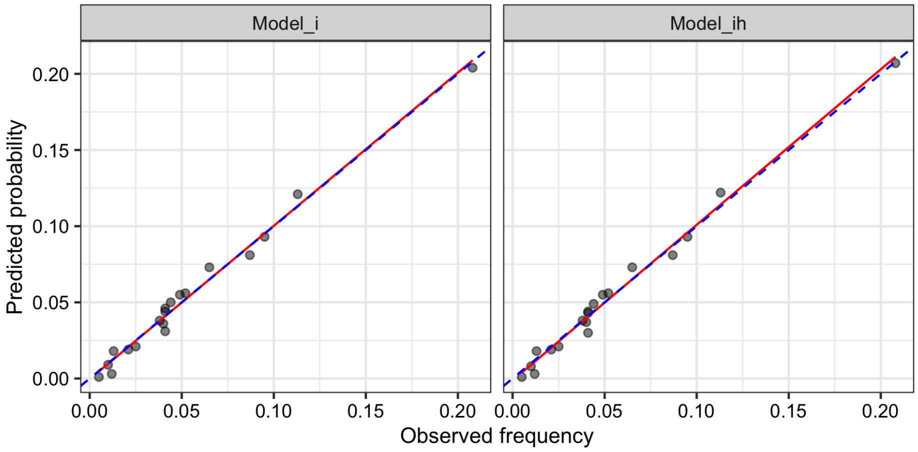

Supplementary Figure S6. Comparison between the observed frequencies of 19 behaviors and the corresponding predicted probabilities obtained by Model_i and Model_ih. The red solid line represents the best-fit line and the blue dashed line represents the diagonal line.

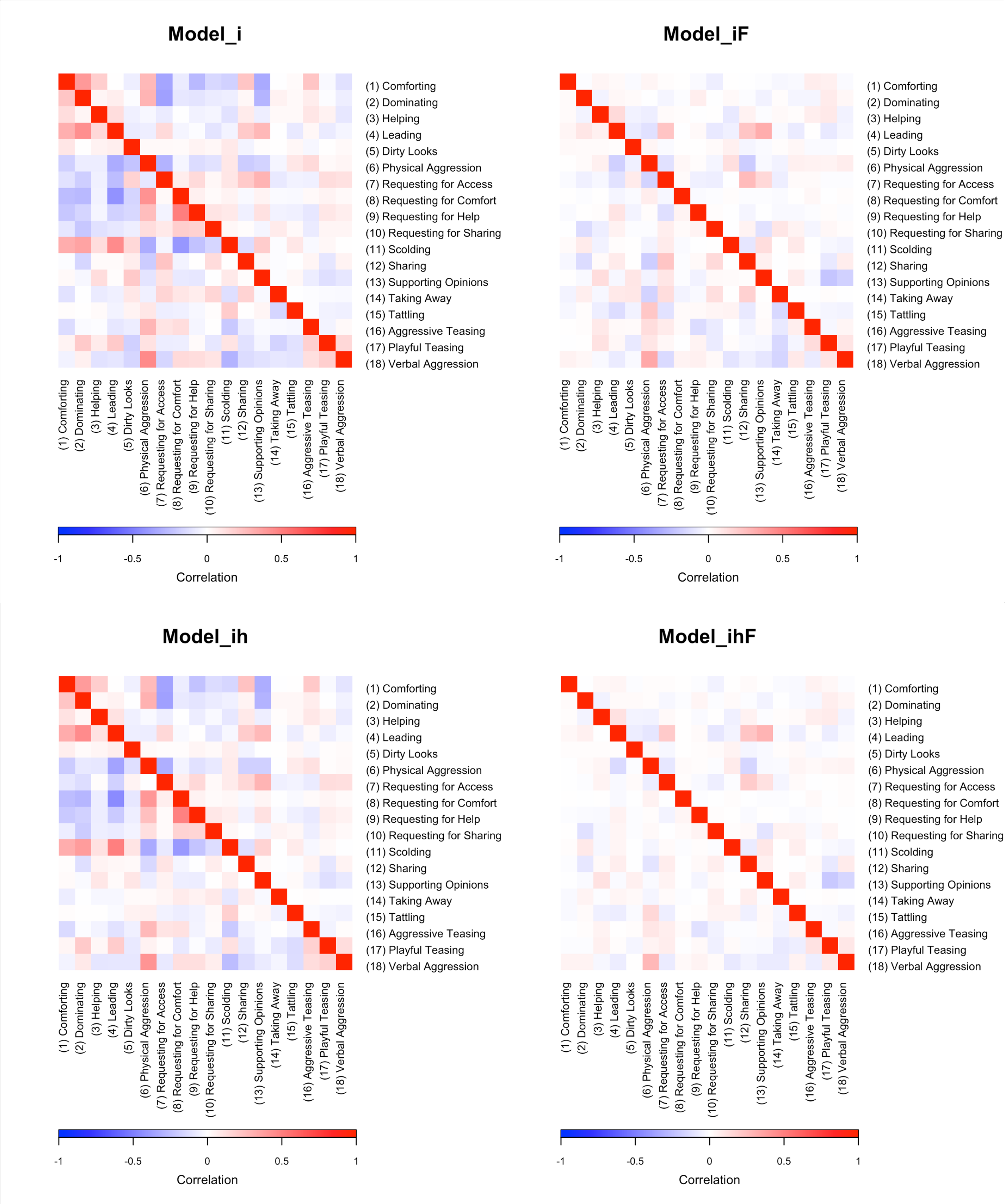

Supplementary Figure S7. Heatmaps of the posterior correlations of individual-level random effects across behaviors obtained by Model_i (top left panel), Model_iF (top right panel), Model_ih (bottom left panel), and Model_ihF (bottom right panel). The bottom half of each heatmap depicts correlations of random effects from initiators. The top half of each heatmap depicts correlations of random effects from recipients.

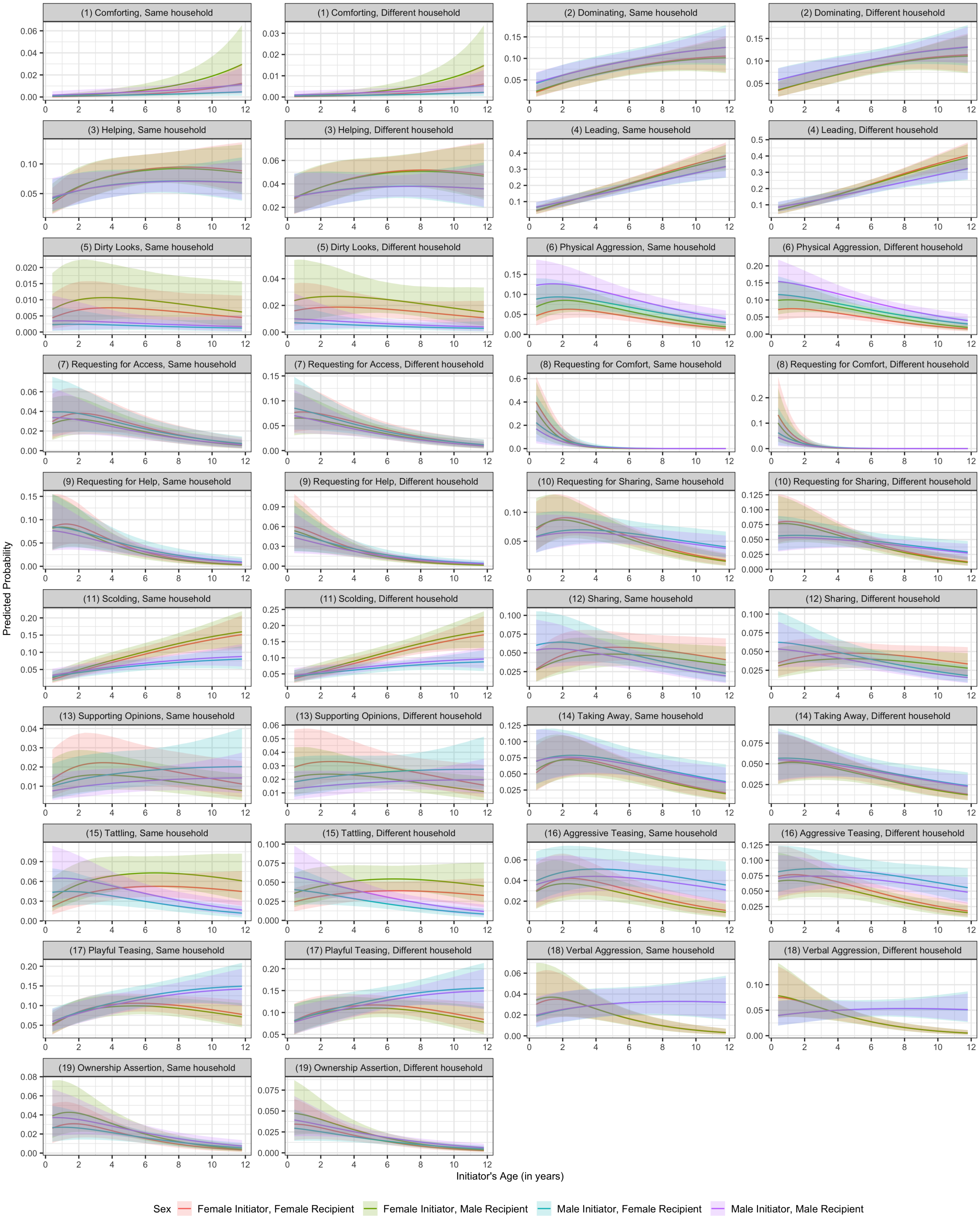

Supplementary Figure S8. Predicted probabilities of response behaviors as a function of initiator's age. The recipient's age is held at the sample mean. Four separate probabilities curves are calculated according to different combinations of sexes, where initiator's sex and recipient's sex are held constant for each curve. The coefficients of fixed effects used in the prediction are derived from Model_iF, as listed in Supplementary Table S6. The shaded regions are the 95% percentile intervals, as calculated from the posterior samples of Model_iF.

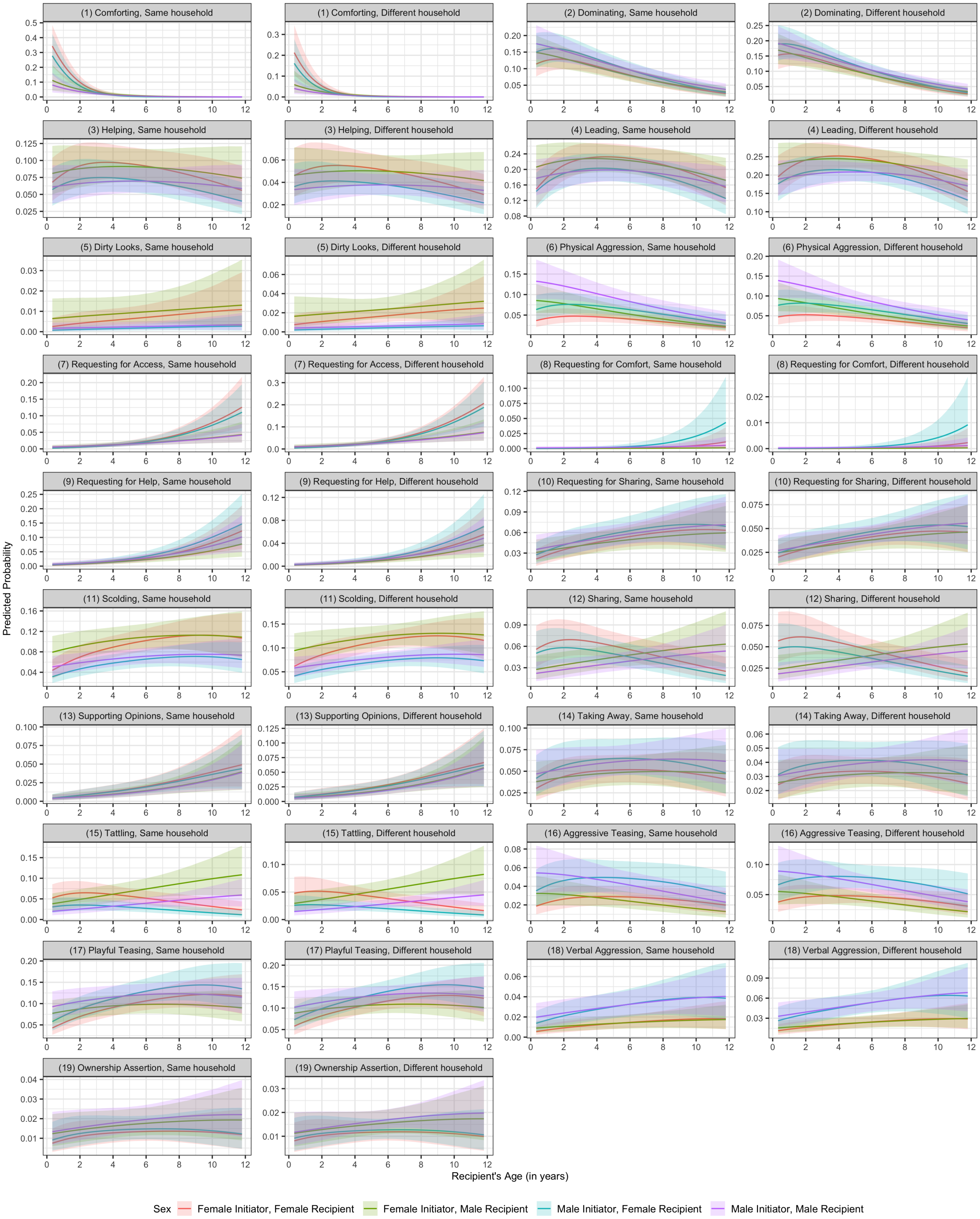

Supplementary Figure S9. Predicted probabilities of response behaviors as a function of recipient's age. The initiator's age is held at the sample mean. Four separate probabilities curves are calculated according to different combinations of sexes, where initiator's sex and recipient's sex are held constant for each curve. The coefficients of fixed effects used in the prediction are derived from Model_iF, as listed in Supplementary Table S6. The shaded regions are the 95% percentile intervals, as calculated from the posterior samples of Model_iF.

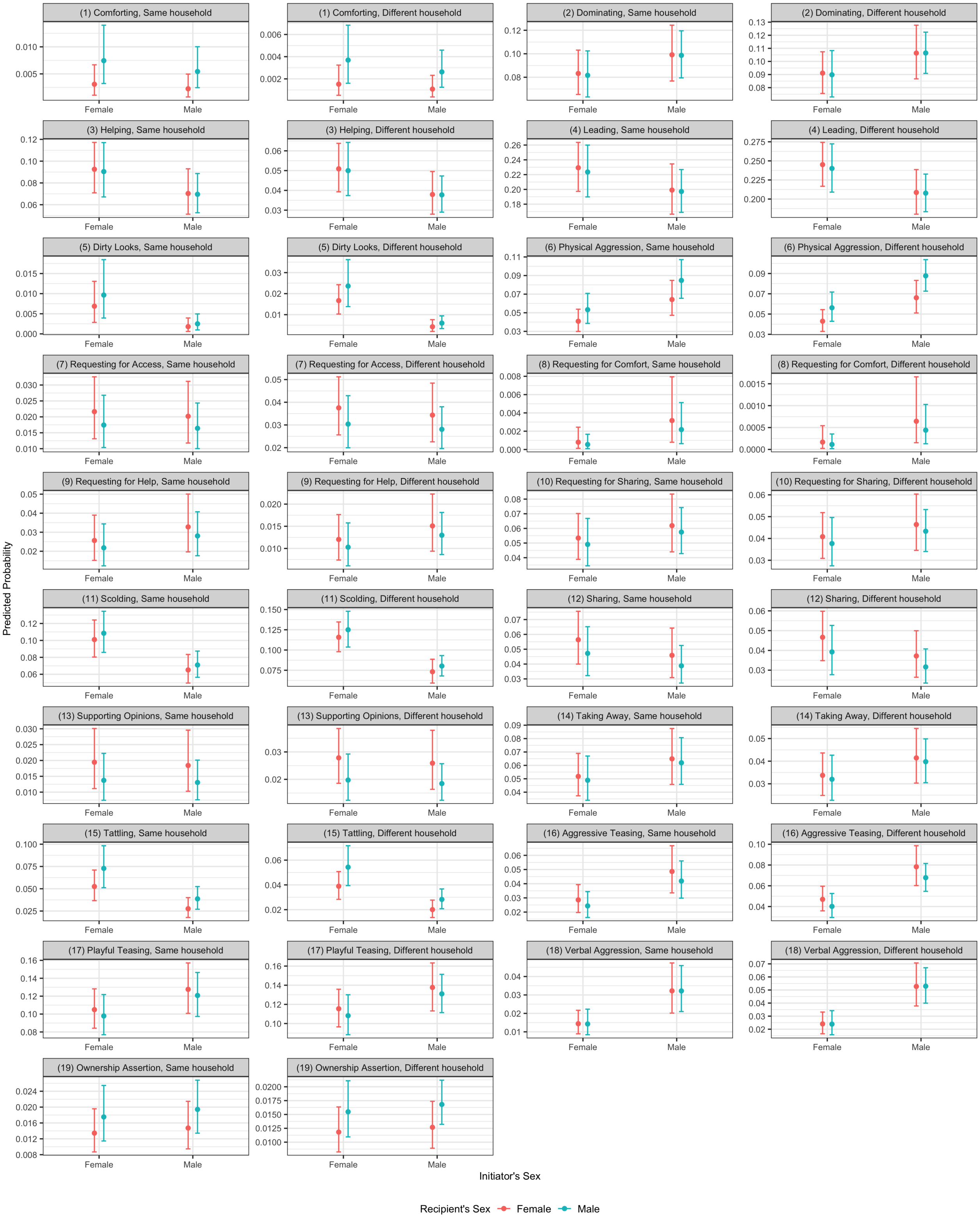

Supplementary Figure S10. Predicted probabilities of response behaviors as a function of initiator's sex. All continuous covariates are held constant at the sample mean. The confidence intervals are the 95% percentile intervals, as calculated from the posterior samples of Model_iF. The coefficients of fixed effects used in the prediction are listed in Supplementary Table S6.

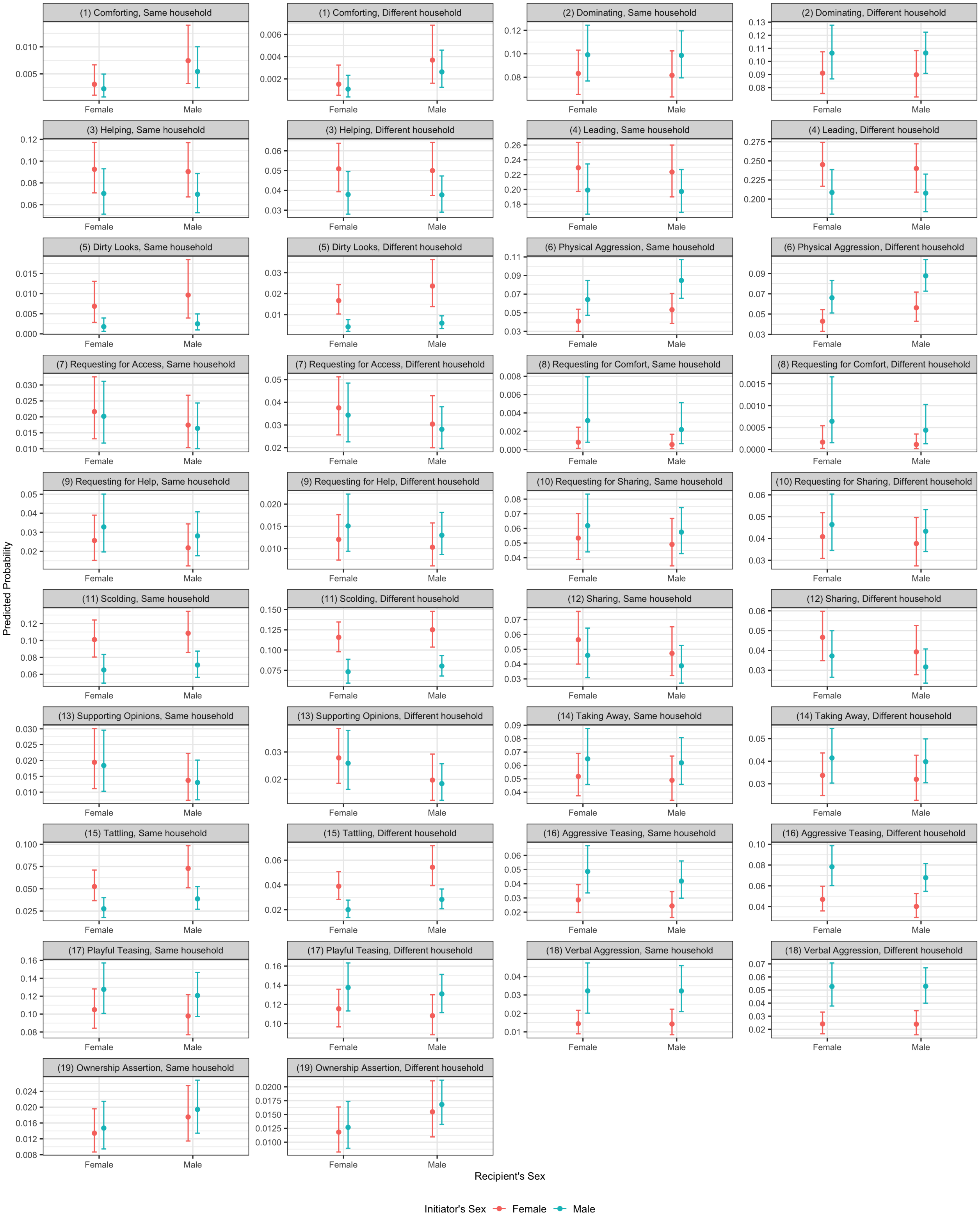

Supplementary Figure S11. Predicted probabilities of response behaviors as a function of recipient's sex. All continuous covariates are held constant at the sample mean. The confidence intervals are the 95% percentile intervals, as calculated from the posterior samples of Model_iF. The coefficients of fixed effects used in the prediction are listed in Supplementary Table S6.

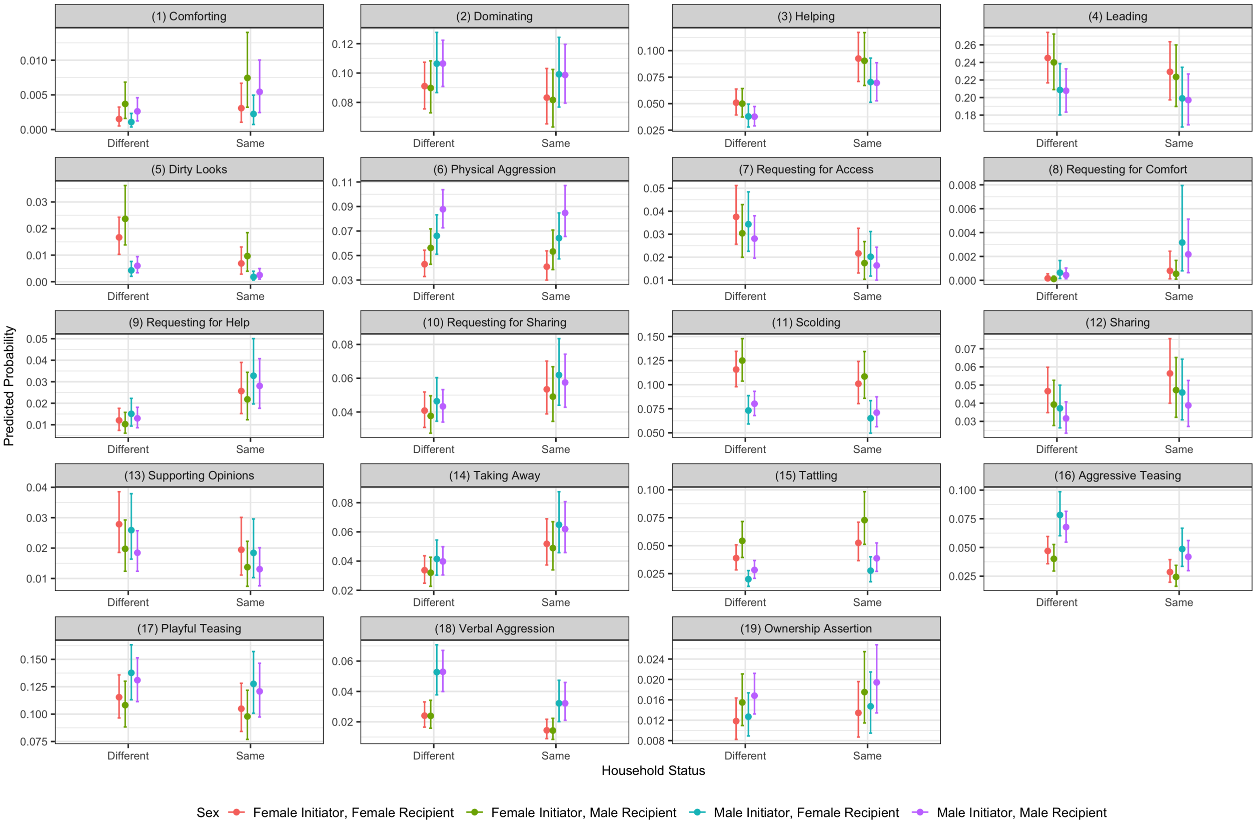

Supplementary Figure S12. Predicted probabilities of response behaviors as a function of household status of the initiator and the recipient. All continuous covariates are held constant at the sample mean. The confidence intervals are the 95% percentile intervals, as calculated from the posterior samples of Model_iF. The coefficients of fixed effects used in the prediction are listed in Supplementary Table S6.

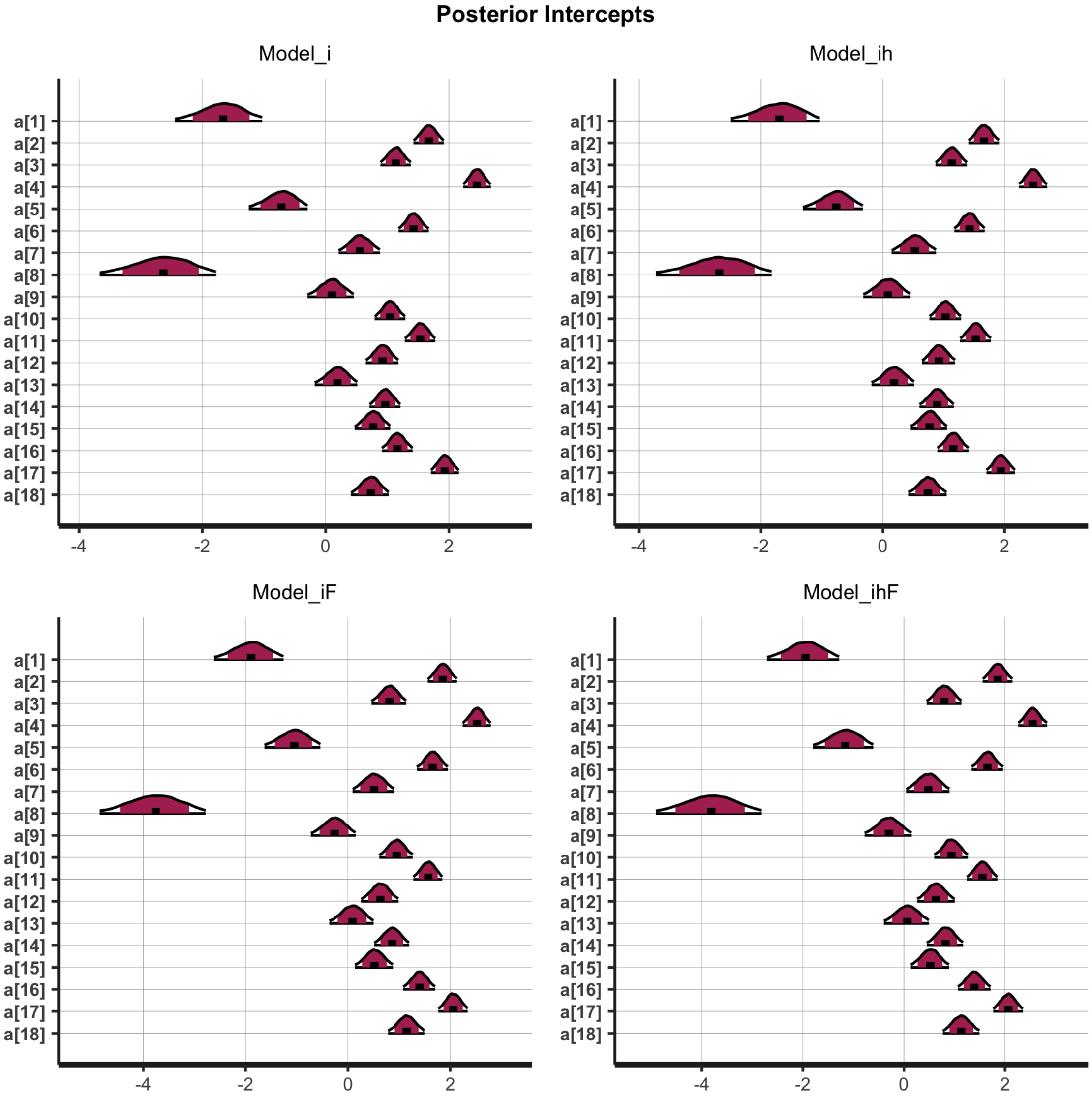

Supplementary Figure S13. Posterior intervals (black horizontal intervals) and posterior median (black ticks on the intervals) of intercepts, α_k, obtained from four fitted models. Kernel density estimates are plotted above the intervals, with the 80% credible interval being shaded.

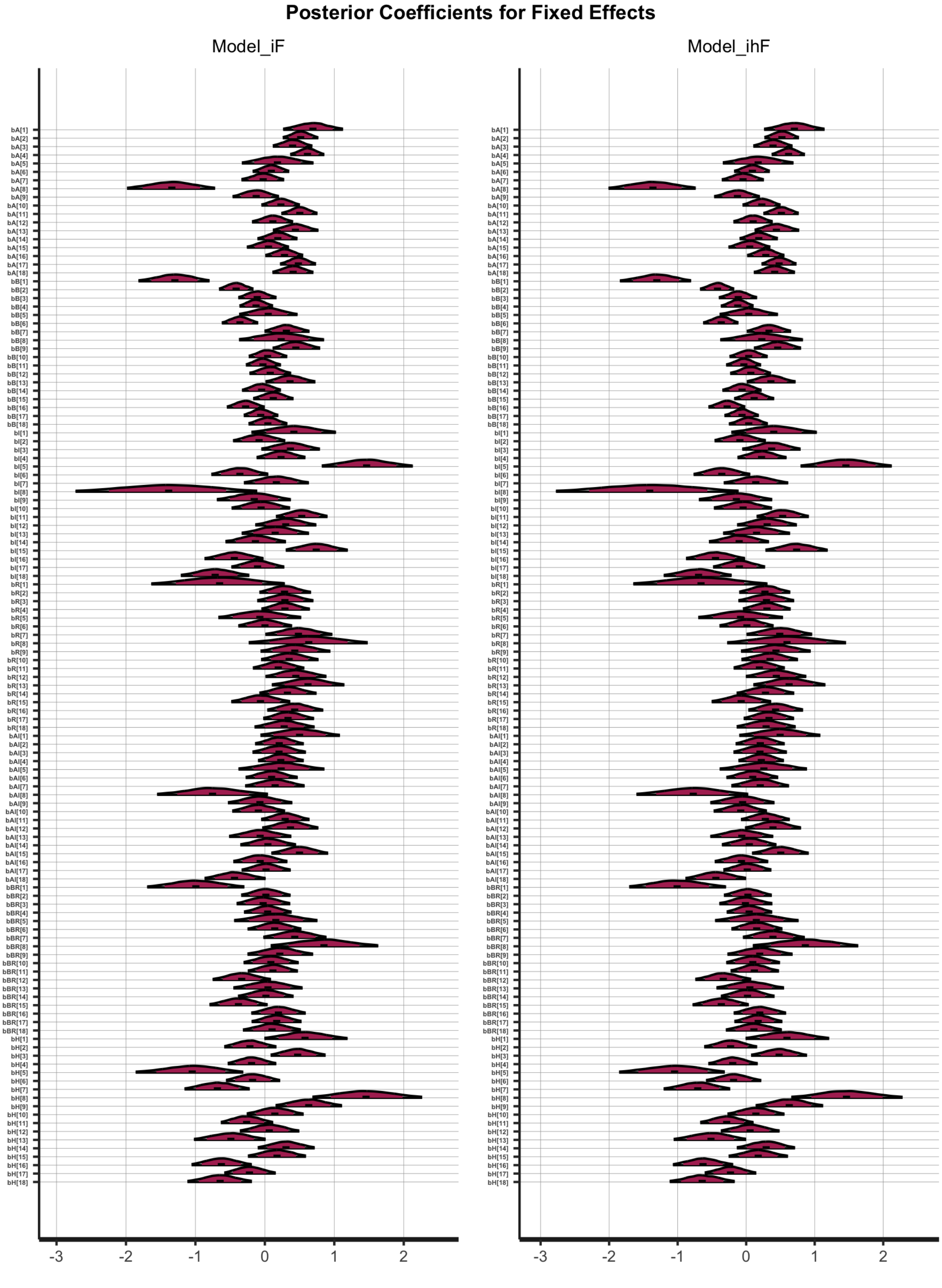

Supplementary Figure S14. Posterior intervals (black horizontal intervals) and posterior median (black ticks on the intervals) of fixed effect coefficients, $\beta_{k,Im}$, $\beta_{k,Rm}$, $\beta_{k,H}$, obtained from two fitted models. Kernel density estimates are plotted above the intervals, with the 80% credible interval being shaded. *bA* coefficient of initiator's age. *bB* coefficient of recipient's age. *bI* coefficient of initiator's sex. *bR* coefficient of recipient's sex. *bAI* coefficient of interaction between initiator's age and sex. *bBR* coefficient of interaction between recipient's age and sex. *bH* coefficient of same household.

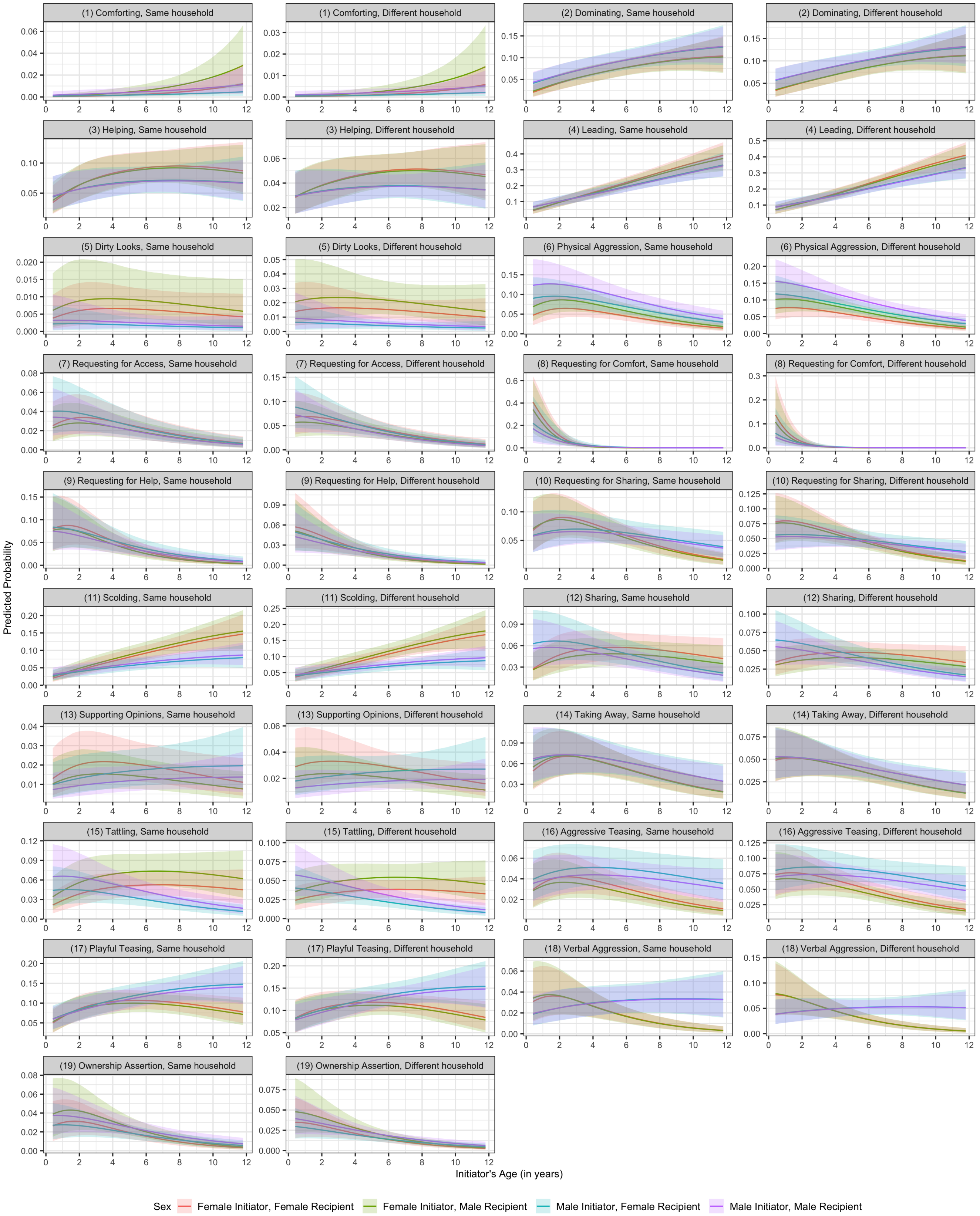

Supplementary Figure S15. Predicted probabilities of response behaviors as a function of initiator's age. The recipient's age is held at the sample mean. Four separate probabilities curves are calculated according to different combinations of sexes, where initiator's sex and recipient's sex are held constant for each curve. The shaded regions are the 95% percentile intervals, as calculated from the posterior samples of Model_ihF. The coefficients of fixed effects used in the prediction are listed in Supplemental Table S7.

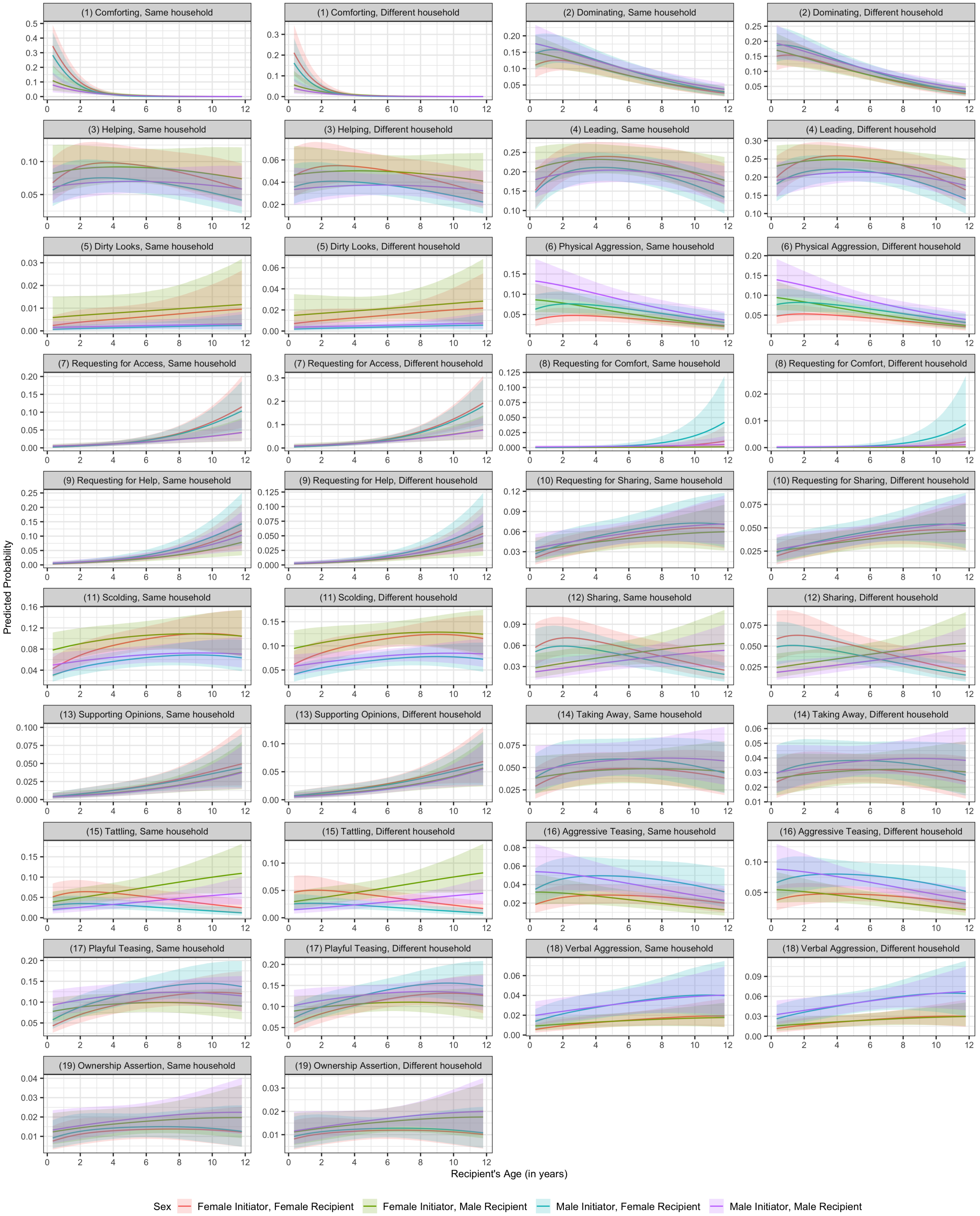

Supplementary Figure S16. Predicted probabilities of response behaviors as a function of recipient's age. The initiator's age is held at the sample mean. Four separate probabilities curves are calculated according to different combinations of sexes, where initiator's sex and recipient's sex are held constant for each curve. The shaded regions are the 95% percentile intervals, as calculated from the posterior samples of Model_ihF. The coefficients of fixed effects used in the prediction are listed in Supplementary Table S7.

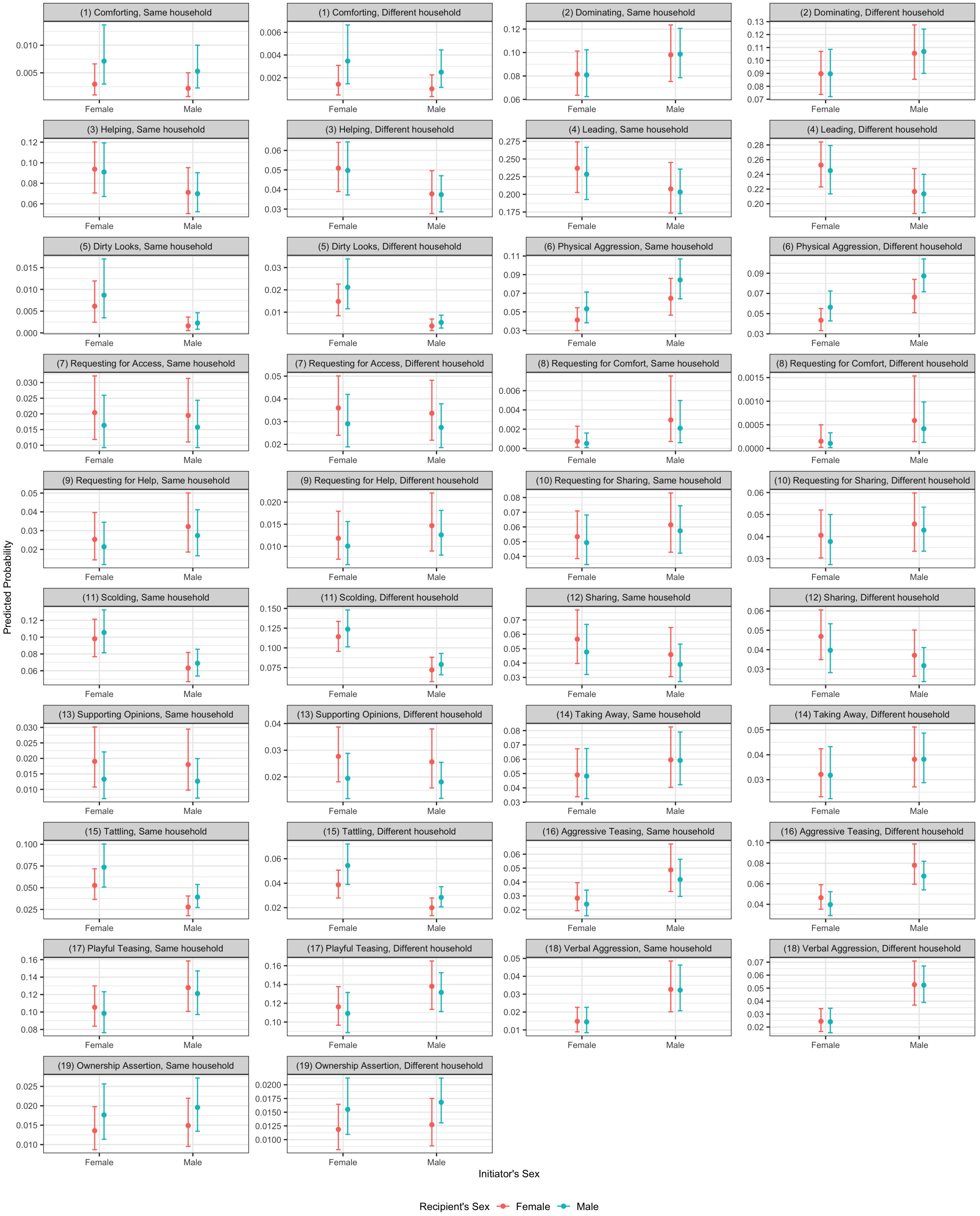

Supplementary Figure S17. Predicted probabilities of response behaviors as a function of initiator's sex. All continuous covariates are held constant at the sample mean. The confidence intervals are the 95% percentile intervals, as calculated from the posterior samples of Model_ihF. The coefficients of fixed effects used in the prediction are listed in Supplementary Table S7.

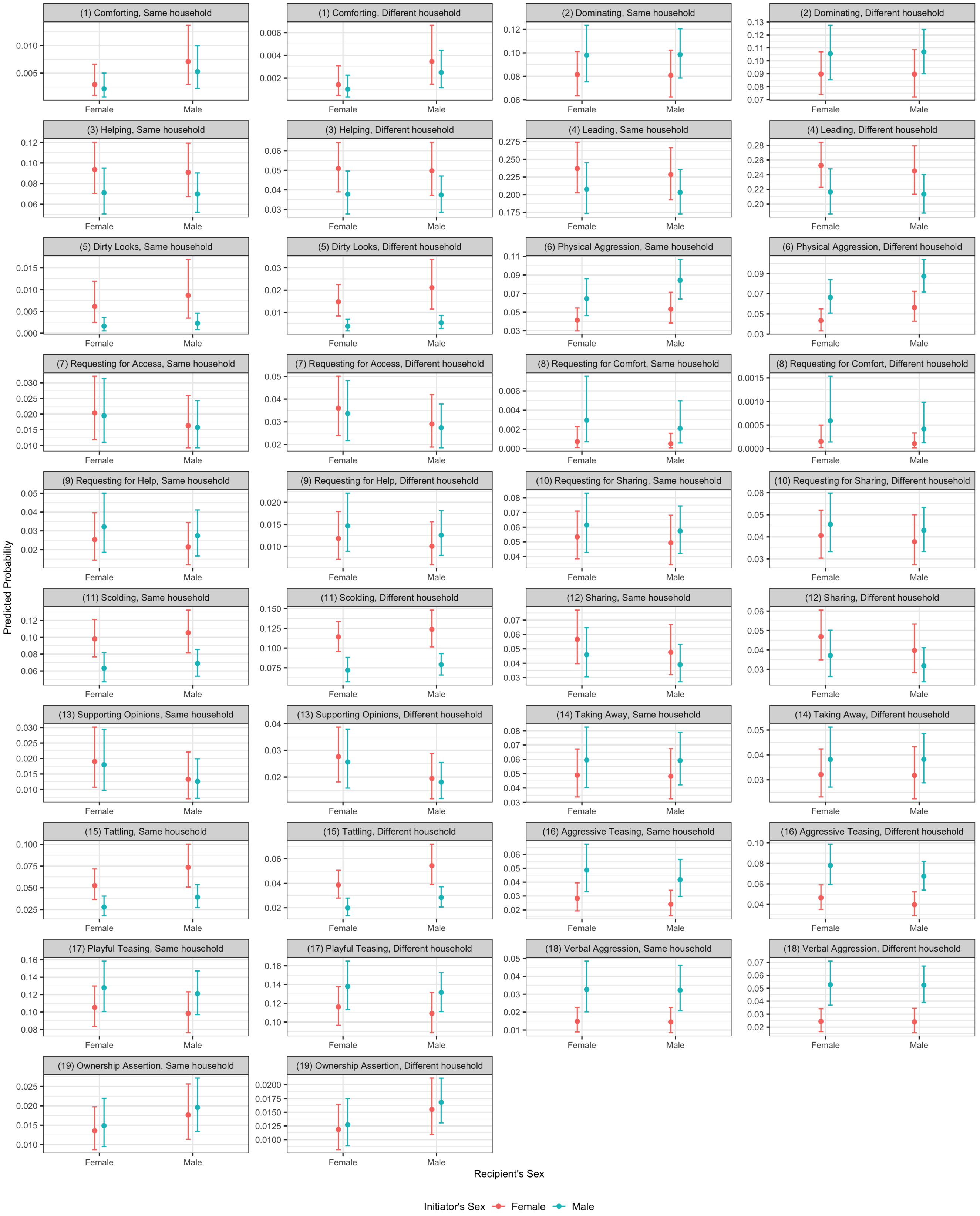

Supplementary Figure S18. Predicted probabilities of response behaviors as a function of recipient's sex. All continuous covariates are held constant at the sample mean. The confidence intervals are the 95% percentile intervals, as calculated from the posterior samples of Model_ihF. The coefficients of fixed effects used in the prediction are listed in Supplementary Table S7.

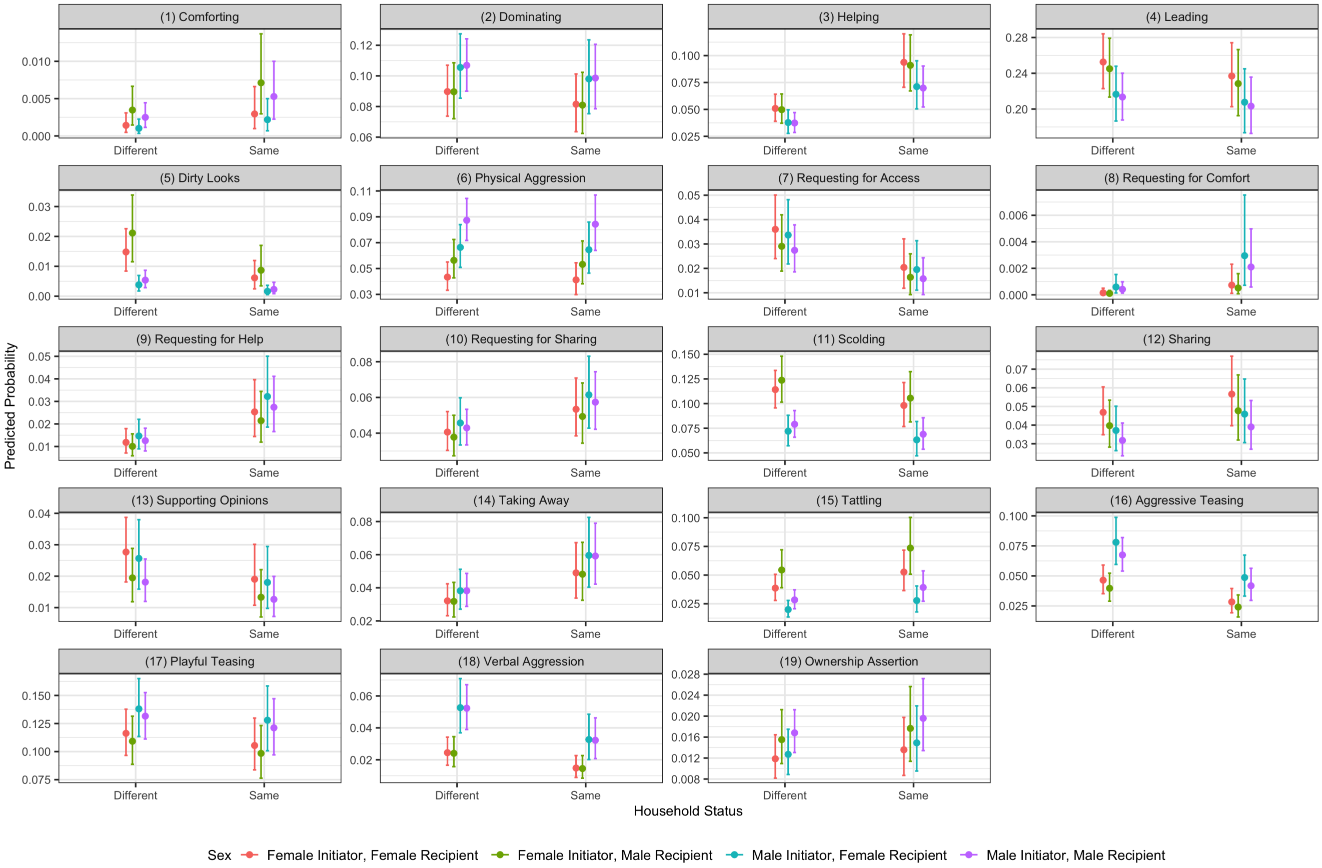

Supplementary Figure S19. Predicted probabilities of response behaviors as a function of household status of the initiator and the recipient. All continuous covariates are held constant at the sample mean. The confidence intervals are the 95% percentile intervals, as calculated from the posterior samples of Model_ihF. The coefficients of fixed effects used in the prediction are listed in Supplementary Table S7.

Supplementary Table

Behavior Index	Behavior Description	Number of Occurrences	Examples or Clarifications
1	Comforting	77	Making others feel better, consoling, offering emotional support
2	Dominating	618	Exerting control over others, demanding obedience, insisting on one's own way
3	Helping	322	Assisting others with tasks or problems, providing advice or information, defending or protecting others
4	Leading	1356	Initiating or directing others in a collaborative context, coordinating group activities
5	Dirty Looks	63	Giving others negative or disapproving facial expressions
6	Physical Aggression	420	Harming or attempting to harm others physically, using force or violence
7	Requesting for Access	267	Asking to join a group or activity, requesting a turn or opportunity to participate
8	Requesting for Comfort	32	Asking for comfort or emotional support from others
9	Requesting for Help	137	Asking for assistance or aid from others
10	Requesting for Sharing	286	Asking for resources or items from others
11	Scolding	567	Criticizing others harshly or repeatedly, using verbal aggression or intimidation
12	Sharing	269	Offering resources or items to others, promoting fairness or cooperation
13	Supporting Opinions	164	Agreeing with or validating others' perspectives or ideas
14	Taking Away	270	Removing items or resources from others without permission, acting selfishly or possessively
15	Tattling	246	Reporting or informing authority figures about others' behavior or actions, seeking to get others in trouble
16	Aggressive Teasing	336	Taunting, teasing, or provoking others in a hostile or aggressive manner
17	Playful Teasing	734	Teasing or joking with others in a lighthearted or friendly manner
18	Verbal Aggression	259	Insulting, cursing, or using hostile language toward others
19	Ownership Assertion	84	Asserting one's rights or control over possessions or resources

Supplementary Table S1. Types of Proactive Social Behaviors Observed in the Study. This table shows the different types of social behaviors recorded during observations of participants in the study. Each behavior is identified by a unique index and described in terms of its typical features and examples. The number of occurrences of each behavior is also provided.

	(1)	(2)	(3)	(4)	(5)	(6)	(7)	(8)	(9)	(10)	(11)	(12)	(13)	(14)	(15)	(16)	(17)	(18)
(1)	1	**0.393 (0.154)**	0.192 (0.203)	0.001 (0.181)	-0.062 (0.216)	0.270 (0.168)	**-0.351 (0.146)**	-0.078 (0.219)	-0.269 (0.189)	-0.150 (0.207)	-0.173 (0.201)	0.221 (0.173)	-0.328 (0.159)	-0.003 (0.207)	0.017 (0.197)	0.225 (0.199)	-0.026 (0.180)	-0.117 (0.198)
(2)	0.224 (0.197)	1	0.039 (0.197)	0.063 (0.184)	-0.009 (0.216)	0.291 (0.171)	**-0.370 (0.147)**	-0.092 (0.218)	-0.104 (0.187)	-0.079 (0.198)	-0.062 (0.199)	0.031 (0.180)	-0.290 (0.161)	0.053 (0.211)	0.022 (0.199)	0.073 (0.198)	-0.007 (0.180)	-0.064 (0.194)
(3)	0.129 (0.205)	0.040 (0.187)	1	0.127 (0.206)	-0.057 (0.219)	-0.032 (0.202)	-0.080 (0.192)	-0.004 (0.214)	-0.051 (0.205)	-0.035 (0.212)	-0.112 (0.212)	0.074 (0.204)	-0.067 (0.196)	0.022 (0.215)	0.023 (0.210)	0.098 (0.216)	0.050 (0.203)	-0.090 (0.209)
(4)	0.311 (0.196)	**0.438 (0.140)**	0.148 (0.184)	1	-0.078 (0.220)	-0.060 (0.186)	0.144 (0.167)	-0.036 (0.215)	-0.008 (0.191)	-0.102 (0.204)	-0.042 (0.200)	0.201 (0.183)	0.278 (0.171)	-0.004 (0.211)	-0.081 (0.202)	0.027 (0.201)	-0.079 (0.189)	-0.035 (0.198)
(5)	0.094 (0.205)	0.005 (0.188)	0.052 (0.203)	0.065 (0.187)	1	0.022 (0.213)	0.023 (0.212)	0.014 (0.218)	0.040 (0.218)	0.028 (0.219)	0.084 (0.224)	-0.043 (0.216)	0.007 (0.214)	0.006 (0.215)	0.030 (0.216)	-0.032 (0.220)	0.005 (0.212)	0.025 (0.218)
(6)	-0.182 (0.193)	-0.068 (0.167)	-0.068 (0.187)	**-0.321 (0.150)**	-0.232 (0.193)	1	-0.298 (0.164)	-0.039 (0.216)	-0.138 (0.190)	-0.112 (0.201)	0.092 (0.202)	-0.198 (0.180)	-0.173 (0.174)	-0.038 (0.213)	0.093 (0.206)	0.144 (0.202)	0.023 (0.187)	0.036 (0.199)
(7)	-0.108 (0.193)	-0.188 (0.165)	-0.054 (0.189)	-0.257 (0.157)	0.167 (0.191)	-0.089 (0.172)	1	0.072 (0.215)	0.140 (0.179)	0.030 (0.195)	0.096 (0.197)	0.203 (0.167)	0.297 (0.150)	-0.084 (0.210)	-0.049 (0.194)	-0.055 (0.190)	0.131 (0.167)	0.118 (0.189)
(8)	-0.258 (0.201)	-0.268 (0.170)	-0.048 (0.201)	**-0.426 (0.146)**	-0.162 (0.204)	**0.422 (0.156)**	0.024 (0.188)	1	0.056 (0.215)	0.017 (0.218)	0.041 (0.218)	-0.026 (0.215)	0.041 (0.217)	-0.016 (0.218)	-0.021 (0.218)	0.025 (0.216)	0.041 (0.214)	0.022 (0.217)
(9)	-0.217 (0.199)	-0.161 (0.169)	-0.037 (0.196)	-0.178 (0.160)	-0.147 (0.200)	0.260 (0.166)	-0.040 (0.181)	**0.501 (0.151)**	1	0.100 (0.210)	0.083 (0.212)	-0.055 (0.194)	0.117 (0.189)	-0.047 (0.212)	-0.057 (0.208)	-0.087 (0.206)	0.088 (0.193)	0.063 (0.203)
(10)	-0.096 (0.210)	-0.217 (0.197)	-0.113 (0.207)	-0.090 (0.194)	-0.062 (0.206)	0.131 (0.199)	0.082 (0.198)	0.190 (0.207)	0.178 (0.204)	1	0.033 (0.212)	0.013 (0.203)	-0.038 (0.199)	0.078 (0.220)	0.026 (0.211)	-0.079 (0.213)	0.035 (0.206)	0.034 (0.209)
(11)	0.315 (0.189)	**0.358 (0.152)**	0.129 (0.187)	**0.451 (0.136)**	0.152 (0.186)	**-0.322 (0.154)**	-0.035 (0.165)	**-0.402 (0.162)**	-0.239 (0.166)	-0.158 (0.195)	1	-0.136 (0.205)	0.197 (0.205)	-0.010 (0.216)	0.087 (0.214)	-0.025 (0.210)	0.030 (0.205)	0.002 (0.209)
(12)	-0.011 (0.202)	-0.158 (0.186)	0.020 (0.203)	0.014 (0.182)	0.067 (0.201)	-0.195 (0.189)	0.233 (0.193)	-0.041 (0.200)	0.003 (0.196)	0.134 (0.211)	0.000 (0.186)	1	0.035 (0.177)	-0.013 (0.209)	-0.003 (0.200)	0.036 (0.202)	-0.097 (0.185)	0.041 (0.202)
(13)	0.027 (0.210)	-0.033 (0.198)	0.132 (0.216)	0.010 (0.196)	0.208 (0.222)	-0.105 (0.200)	0.108 (0.203)	-0.081 (0.210)	-0.093 (0.204)	-0.001 (0.213)	0.081 (0.201)	0.010 (0.209)	1	-0.012 (0.207)	0.027 (0.198)	-0.070 (0.198)	-0.125 (0.179)	-0.092 (0.195)
(14)	-0.110 (0.205)	-0.006 (0.187)	-0.078 (0.200)	-0.055 (0.180)	0.092 (0.203)	-0.055 (0.186)	0.162 (0.193)	0.099 (0.195)	0.068 (0.195)	0.154 (0.213)	-0.126 (0.183)	0.167 (0.206)	-0.023 (0.208)	1	-0.005 (0.216)	-0.009 (0.214)	-0.014 (0.211)	-0.020 (0.217)
(15)	0.012 (0.197)	0.001 (0.175)	0.014 (0.193)	-0.042 (0.166)	0.043 (0.195)	0.089 (0.175)	-0.066 (0.178)	-0.056 (0.193)	-0.030 (0.184)	0.008 (0.202)	0.195 (0.166)	0.000 (0.195)	0.039 (0.205)	-0.163 (0.194)	1	-0.008 (0.211)	-0.048 (0.203)	-0.054 (0.210)
(16)	-0.138 (0.196)	-0.016 (0.176)	-0.013 (0.192)	-0.090 (0.167)	-0.068 (0.196)	0.283 (0.168)	0.104 (0.176)	0.132 (0.185)	-0.039 (0.183)	0.006 (0.200)	-0.210 (0.168)	-0.070 (0.193)	-0.018 (0.202)	-0.024 (0.193)	-0.031 (0.183)	1	-0.046 (0.201)	0.023 (0.210)
(17)	0.044 (0.199)	0.205 (0.173)	0.074 (0.193)	0.201 (0.166)	-0.186 (0.197)	0.111 (0.175)	-0.069 (0.177)	-0.062 (0.188)	0.014 (0.179)	-0.093 (0.197)	0.100 (0.172)	-0.116 (0.192)	-0.039 (0.204)	-0.173 (0.188)	-0.108 (0.179)	0.184 (0.177)	1	0.151 (0.202)
(18)	-0.047 (0.188)	0.056 (0.159)	-0.111 (0.184)	-0.095 (0.151)	-0.159 (0.187)	**0.456 (0.142)**	-0.156 (0.162)	0.142 (0.172)	0.128 (0.166)	0.104 (0.193)	**-0.297 (0.147)**	-0.136 (0.185)	-0.107 (0.200)	-0.087 (0.181)	-0.107 (0.169)	0.122 (0.169)	0.192 (0.164)	1

Supplementary Table S2. Correlations of individual-level random effects across behaviors obtained by Model_i. The reported means are from the posterior samples (standard deviation in parentheses). Parameters in bold represent estimates whose 95% credible intervals do not include zero. The bottom half of the matrix depicts correlations of random effects from initiators. The top half of the matrix details correlations of random effects from recipients. Supplementary Fig. S7 provides the visualization of correlations.

	(1)	(2)	(3)	(4)	(5)	(6)	(7)	(8)	(9)	(10)	(11)	(12)	(13)	(14)	(15)	(16)	(17)	(18)
(1)	1	-0.008 (0.219)	0.036 (0.215)	0.024 (0.208)	-0.016 (0.219)	0.034 (0.215)	0.007 (0.213)	0.004 (0.218)	-0.014 (0.214)	-0.061 (0.219)	0.006 (0.217)	-0.003 (0.215)	-0.007 (0.212)	-0.031 (0.215)	-0.024 (0.218)	0.072 (0.219)	0.058 (0.215)	-0.014 (0.213)
(2)	0.019 (0.214)	1	-0.043 (0.215)	0.031 (0.207)	0.015 (0.219)	0.000 (0.210)	-0.040 (0.207)	-0.010 (0.216)	0.063 (0.218)	-0.005 (0.215)	0.020 (0.213)	-0.008 (0.207)	-0.047 (0.207)	0.010 (0.213)	-0.035 (0.215)	-0.037 (0.216)	0.074 (0.207)	0.029 (0.213)
(3)	0.015 (0.216)	-0.041 (0.199)	1	0.140 (0.208)	-0.049 (0.219)	-0.066 (0.214)	0.020 (0.202)	0.007 (0.217)	0.000 (0.212)	0.003 (0.214)	-0.084 (0.214)	0.049 (0.206)	-0.008 (0.201)	0.023 (0.217)	0.012 (0.214)	0.064 (0.215)	0.081 (0.207)	-0.048 (0.212)
(4)	0.039 (0.216)	0.096 (0.195)	0.055 (0.189)	1	-0.076 (0.217)	-0.118 (0.198)	0.232 (0.167)	-0.004 (0.215)	0.024 (0.208)	-0.084 (0.202)	-0.055 (0.204)	0.226 (0.178)	0.317 (0.167)	-0.021 (0.208)	-0.075 (0.204)	0.006 (0.203)	-0.081 (0.185)	-0.039 (0.199)
(5)	0.023 (0.222)	0.019 (0.212)	0.013 (0.212)	0.010 (0.207)	1	0.037 (0.217)	-0.019 (0.213)	0.003 (0.217)	0.001 (0.215)	0.018 (0.215)	0.058 (0.222)	-0.036 (0.216)	-0.016 (0.215)	0.011 (0.218)	0.028 (0.217)	-0.019 (0.220)	-0.018 (0.214)	0.006 (0.217)
(6)	0.003 (0.214)	0.015 (0.195)	0.055 (0.193)	-0.181 (0.181)	-0.057 (0.212)	1	-0.119 (0.199)	0.028 (0.217)	0.010 (0.215)	-0.034 (0.211)	0.120 (0.221)	-0.194 (0.205)	-0.022 (0.201)	-0.047 (0.214)	0.053 (0.213)	0.048 (0.214)	0.046 (0.205)	0.067 (0.215)
(7)	-0.018 (0.215)	-0.058 (0.195)	-0.030 (0.188)	-0.113 (0.179)	0.075 (0.211)	-0.171 (0.183)	1	0.019 (0.218)	-0.055 (0.207)	-0.063 (0.203)	-0.003 (0.205)	0.262 (0.174)	0.147 (0.172)	-0.065 (0.209)	0.015 (0.200)	0.017 (0.204)	-0.007 (0.179)	-0.012 (0.199)
(8)	0.000 (0.221)	-0.009 (0.215)	0.001 (0.214)	-0.034 (0.217)	-0.017 (0.217)	0.073 (0.222)	-0.028 (0.217)	1	-0.017 (0.218)	-0.008 (0.216)	0.009 (0.219)	-0.008 (0.218)	-0.008 (0.217)	-0.017 (0.220)	0.001 (0.216)	0.027 (0.219)	-0.003 (0.217)	0.012 (0.219)
(9)	-0.015 (0.218)	0.005 (0.208)	0.009 (0.203)	0.103 (0.202)	-0.042 (0.217)	0.009 (0.207)	-0.125 (0.206)	0.040 (0.217)	1	0.010 (0.215)	-0.004 (0.216)	-0.037 (0.208)	-0.002 (0.207)	-0.024 (0.218)	-0.020 (0.213)	-0.010 (0.214)	0.007 (0.209)	-0.010 (0.215)
(10)	-0.001 (0.220)	-0.135 (0.214)	-0.062 (0.207)	0.053 (0.205)	-0.011 (0.215)	-0.009 (0.200)	0.064 (0.205)	0.016 (0.216)	0.043 (0.215)	1	-0.003 (0.214)	0.043 (0.208)	-0.113 (0.201)	0.096 (0.225)	0.041 (0.212)	-0.039 (0.216)	0.032 (0.206)	0.007 (0.211)
(11)	0.015 (0.217)	0.076 (0.214)	-0.047 (0.212)	0.012 (0.209)	0.000 (0.217)	-0.042 (0.206)	0.079 (0.209)	-0.014 (0.217)	-0.005 (0.213)	0.001 (0.212)	1	-0.097 (0.208)	0.130 (0.211)	0.001 (0.215)	0.081 (0.219)	-0.001 (0.216)	-0.032 (0.207)	-0.043 (0.215)
(12)	-0.033 (0.216)	-0.136 (0.202)	0.014 (0.201)	0.079 (0.192)	0.003 (0.212)	-0.188 (0.197)	0.223 (0.196)	-0.010 (0.219)	0.026 (0.209)	0.145 (0.215)	0.005 (0.211)	1	0.059 (0.180)	-0.005 (0.207)	0.007 (0.203)	0.006 (0.205)	-0.082 (0.186)	0.072 (0.204)
(13)	0.006 (0.216)	-0.023 (0.211)	0.127 (0.213)	-0.027 (0.203)	0.097 (0.224)	-0.046 (0.209)	0.094 (0.203)	-0.008 (0.218)	-0.052 (0.214)	0.024 (0.213)	0.018 (0.216)	0.009 (0.209)	1	-0.003 (0.206)	0.079 (0.205)	-0.022 (0.205)	-0.221 (0.180)	-0.180 (0.208)
(14)	-0.044 (0.216)	0.061 (0.204)	-0.031 (0.197)	0.084 (0.192)	0.089 (0.220)	-0.171 (0.193)	0.127 (0.193)	-0.042 (0.216)	-0.022 (0.209)	0.125 (0.213)	0.006 (0.210)	0.177 (0.203)	0.000 (0.211)	1	-0.003 (0.215)	-0.004 (0.215)	0.010 (0.211)	-0.006 (0.215)
(15)	-0.031 (0.218)	0.013 (0.201)	-0.062 (0.200)	-0.131 (0.186)	-0.048 (0.212)	0.215 (0.194)	-0.107 (0.193)	0.016 (0.215)	0.025 (0.206)	0.037 (0.207)	0.043 (0.214)	-0.086 (0.199)	0.013 (0.210)	-0.147 (0.201)	1	-0.014 (0.213)	-0.034 (0.204)	-0.045 (0.213)
(16)	0.001 (0.216)	0.012 (0.199)	0.082 (0.196)	0.022 (0.189)	0.014 (0.213)	0.131 (0.196)	0.076 (0.190)	0.019 (0.220)	-0.135 (0.210)	-0.077 (0.207)	-0.006 (0.210)	-0.042 (0.202)	0.015 (0.207)	-0.084 (0.197)	0.061 (0.199)	1	-0.028 (0.206)	0.039 (0.214)
(17)	-0.004 (0.216)	0.002 (0.196)	0.107 (0.192)	0.030 (0.185)	-0.082 (0.216)	0.094 (0.190)	0.029 (0.184)	-0.002 (0.215)	0.042 (0.207)	-0.078 (0.206)	0.009 (0.209)	-0.024 (0.195)	-0.031 (0.207)	-0.151 (0.192)	-0.039 (0.194)	0.136 (0.191)	1	0.101 (0.205)
(18)	0.050 (0.213)	0.029 (0.193)	-0.022 (0.186)	-0.077 (0.182)	-0.025 (0.213)	0.337 (0.174)	-0.145 (0.178)	0.041 (0.217)	0.057 (0.203)	0.022 (0.204)	-0.103 (0.212)	-0.058 (0.192)	-0.094 (0.210)	-0.161 (0.188)	0.083 (0.192)	-0.074 (0.187)	0.103 (0.181)	1

Supplementary Table S3. Correlations of individual-level random effects across behaviors obtained by Model_iF. The reported means are from the posterior samples (standard deviation in parentheses). Parameters in bold represent estimates whose 95% credible intervals do not include zero. The bottom half of the matrix depicts correlations of random effects from initiators. The top half of the matrix details correlations of random effects from recipients.

	(1)	(2)	(3)	(4)	(5)	(6)	(7)	(8)	(9)	(10)	(11)	(12)	(13)	(14)	(15)	(16)	(17)	(18)
(1)	1	**0.392 (0.156)**	0.189 (0.206)	0.003 (0.183)	-0.054 (0.216)	0.262 (0.172)	**-0.357 (0.149)**	-0.072 (0.217)	-0.235 (0.205)	-0.134 (0.209)	-0.158 (0.205)	0.236 (0.179)	-0.317 (0.162)	-0.005 (0.214)	0.004 (0.201)	0.212 (0.200)	-0.026 (0.184)	-0.103 (0.203)
(2)	0.223 (0.196)	1	0.030 (0.198)	0.048 (0.188)	-0.005 (0.216)	0.306 (0.175)	**-0.372 (0.152)**	-0.084 (0.218)	-0.101 (0.196)	-0.077 (0.201)	-0.054 (0.202)	0.041 (0.183)	-0.294 (0.164)	0.031 (0.215)	0.030 (0.202)	0.068 (0.197)	-0.004 (0.187)	-0.047 (0.198)
(3)	0.110 (0.213)	0.028 (0.200)	1	0.112 (0.211)	-0.054 (0.219)	-0.026 (0.204)	-0.065 (0.195)	-0.002 (0.220)	-0.070 (0.208)	-0.027 (0.210)	-0.092 (0.212)	0.084 (0.205)	-0.060 (0.199)	0.010 (0.219)	0.030 (0.214)	0.093 (0.214)	0.049 (0.205)	-0.077 (0.212)
(4)	0.322 (0.199)	**0.448 (0.141)**	0.111 (0.199)	1	-0.065 (0.218)	-0.070 (0.191)	0.150 (0.177)	-0.026 (0.217)	0.006 (0.204)	-0.098 (0.208)	-0.032 (0.209)	0.199 (0.193)	0.269 (0.179)	-0.007 (0.214)	-0.069 (0.208)	0.026 (0.203)	-0.064 (0.193)	-0.022 (0.204)
(5)	0.066 (0.217)	0.014 (0.207)	0.038 (0.211)	0.061 (0.209)	1	0.026 (0.215)	0.007 (0.215)	0.011 (0.220)	0.034 (0.216)	0.025 (0.218)	0.069 (0.222)	-0.045 (0.218)	-0.005 (0.214)	0.006 (0.220)	0.029 (0.216)	-0.033 (0.218)	0.011 (0.215)	0.020 (0.216)
(6)	-0.198 (0.194)	-0.085 (0.172)	-0.088 (0.201)	**-0.354 (0.154)**	-0.123 (0.213)	1	-0.307 (0.168)	-0.043 (0.213)	-0.116 (0.202)	-0.085 (0.204)	0.077 (0.208)	-0.182 (0.187)	-0.181 (0.177)	-0.005 (0.218)	0.077 (0.209)	0.119 (0.205)	0.011 (0.192)	0.020 (0.203)
(7)	-0.089 (0.207)	-0.127 (0.195)	0.009 (0.211)	-0.179 (0.195)	0.040 (0.216)	-0.005 (0.198)	1	0.067 (0.218)	0.148 (0.194)	0.026 (0.198)	0.080 (0.204)	0.154 (0.177)	0.289 (0.156)	-0.057 (0.219)	-0.044 (0.195)	-0.076 (0.198)	0.128 (0.177)	0.126 (0.193)
(8)	-0.257 (0.201)	-0.285 (0.174)	-0.068 (0.208)	**-0.450 (0.148)**	-0.090 (0.214)	**0.405 (0.164)**	0.061 (0.205)	1	0.049 (0.219)	0.019 (0.215)	0.030 (0.217)	-0.026 (0.214)	0.037 (0.217)	-0.005 (0.217)	-0.024 (0.217)	0.019 (0.216)	0.035 (0.216)	0.019 (0.219)
(9)	-0.213 (0.200)	-0.180 (0.172)	-0.067 (0.207)	-0.203 (0.163)	-0.065 (0.210)	0.251 (0.174)	0.018 (0.203)	**0.487 (0.154)**	1	0.093 (0.213)	0.064 (0.214)	-0.049 (0.204)	0.117 (0.199)	-0.017 (0.216)	-0.057 (0.213)	-0.081 (0.215)	0.068 (0.205)	0.052 (0.211)
(10)	-0.073 (0.211)	-0.199 (0.205)	-0.063 (0.215)	-0.079 (0.199)	-0.041 (0.216)	0.122 (0.206)	0.019 (0.209)	0.172 (0.212)	0.165 (0.209)	1	0.033 (0.213)	0.000 (0.205)	-0.039 (0.202)	0.044 (0.220)	0.029 (0.212)	-0.061 (0.214)	0.041 (0.206)	0.025 (0.213)
(11)	0.317 (0.193)	**0.385 (0.150)**	0.118 (0.201)	**0.496 (0.137)**	0.101 (0.212)	**-0.327 (0.159)**	-0.084 (0.194)	**-0.397 (0.163)**	-0.228 (0.165)	-0.131 (0.201)	1	-0.126 (0.209)	0.171 (0.211)	-0.002 (0.219)	0.077 (0.217)	-0.027 (0.213)	0.024 (0.209)	-0.006 (0.211)
(12)	0.012 (0.210)	-0.122 (0.201)	0.044 (0.211)	0.030 (0.200)	0.027 (0.214)	-0.148 (0.205)	0.105 (0.213)	-0.037 (0.209)	-0.003 (0.202)	0.073 (0.214)	-0.002 (0.197)	1	0.021 (0.182)	-0.006 (0.216)	-0.008 (0.204)	0.045 (0.205)	-0.099 (0.192)	0.041 (0.203)
(13)	0.019 (0.212)	-0.017 (0.204)	0.092 (0.222)	0.018 (0.205)	0.099 (0.226)	-0.069 (0.209)	0.048 (0.212)	-0.060 (0.216)	-0.067 (0.212)	0.001 (0.216)	0.062 (0.207)	-0.001 (0.211)	1	-0.008 (0.217)	0.019 (0.200)	-0.059 (0.201)	-0.123 (0.185)	-0.081 (0.199)
(14)	-0.068 (0.214)	-0.005 (0.204)	-0.031 (0.214)	-0.033 (0.206)	0.014 (0.217)	0.018 (0.209)	0.040 (0.214)	0.075 (0.214)	0.051 (0.209)	0.081 (0.220)	-0.089 (0.210)	0.071 (0.217)	-0.023 (0.217)	1	0.001 (0.217)	0.007 (0.217)	-0.003 (0.216)	-0.003 (0.215)
(15)	0.011 (0.195)	0.001 (0.175)	0.003 (0.201)	-0.014 (0.171)	0.047 (0.209)	0.058 (0.184)	-0.052 (0.203)	-0.073 (0.194)	-0.049 (0.189)	0.024 (0.207)	0.185 (0.171)	0.010 (0.203)	0.029 (0.210)	-0.054 (0.213)	1	-0.016 (0.213)	-0.035 (0.206)	-0.049 (0.210)
(16)	-0.146 (0.194)	-0.009 (0.174)	-0.045 (0.205)	-0.108 (0.170)	-0.060 (0.208)	0.267 (0.177)	0.102 (0.200)	0.116 (0.186)	-0.042 (0.182)	0.014 (0.207)	-0.202 (0.171)	-0.053 (0.201)	-0.013 (0.207)	0.001 (0.209)	-0.045 (0.187)	1	-0.045 (0.205)	0.012 (0.211)
(17)	0.037 (0.198)	0.206 (0.174)	0.030 (0.205)	0.196 (0.169)	-0.086 (0.215)	0.051 (0.186)	0.012 (0.200)	-0.086 (0.191)	-0.012 (0.186)	-0.076 (0.206)	0.118 (0.174)	-0.084 (0.204)	-0.016 (0.209)	-0.089 (0.212)	-0.138 (0.185)	0.174 (0.181)	1	0.131 (0.208)
(18)	-0.044 (0.188)	0.081 (0.163)	-0.096 (0.199)	-0.091 (0.157)	-0.090 (0.206)	**0.415 (0.154)**	-0.084 (0.196)	0.127 (0.179)	0.124 (0.172)	0.066 (0.201)	-0.257 (0.157)	-0.127 (0.200)	-0.079 (0.208)	-0.034 (0.208)	-0.129 (0.173)	0.129 (0.176)	0.179 (0.172)	1

Supplementary Table S4. Correlations of individual-level random effects across behaviors obtained by Model_ih. The reported means are from the posterior samples (standard deviation in parentheses). Parameters in bold represent estimates whose 95% credible intervals do not include zero. The bottom half of the matrix depicts correlations of random effects from initiators. The top half of the matrix details correlations of random effects from recipients.

	(1)	(2)	(3)	(4)	(5)	(6)	(7)	(8)	(9)	(10)	(11)	(12)	(13)	(14)	(15)	(16)	(17)	(18)
(1)	1	-0.002 (0.217)	0.031 (0.218)	0.039 (0.215)	-0.012 (0.221)	0.007 (0.216)	0.008 (0.217)	0.001 (0.217)	-0.009 (0.219)	-0.048 (0.216)	0.001 (0.217)	0.014 (0.216)	0.002 (0.218)	-0.014 (0.217)	-0.026 (0.216)	0.05 (0.219)	0.033 (0.219)	-0.013 (0.217)
(2)	0.019 (0.213)	1	-0.048 (0.215)	0.018 (0.211)	0.018 (0.218)	0.013 (0.214)	-0.032 (0.213)	-0.007 (0.216)	0.038 (0.218)	-0.013 (0.215)	0.019 (0.218)	-0.009 (0.212)	-0.046 (0.21)	-0.001 (0.217)	-0.021 (0.215)	-0.03 (0.214)	0.06 (0.211)	0.037 (0.214)
(3)	0.008 (0.218)	-0.046 (0.203)	1	0.116 (0.208)	-0.042 (0.222)	-0.049 (0.214)	0.026 (0.206)	0.006 (0.217)	-0.014 (0.215)	0.006 (0.212)	-0.059 (0.217)	0.05 (0.205)	-0.013 (0.202)	0.012 (0.218)	0.02 (0.213)	0.061 (0.217)	0.078 (0.21)	-0.044 (0.213)
(4)	0.027 (0.219)	0.058 (0.207)	-0.002 (0.208)	1	-0.068 (0.218)	-0.099 (0.208)	0.206 (0.188)	0.002 (0.217)	0.017 (0.215)	-0.091 (0.206)	-0.043 (0.211)	0.223 (0.187)	0.293 (0.181)	-0.014 (0.212)	-0.066 (0.207)	0.009 (0.209)	-0.071 (0.19)	-0.025 (0.204)
(5)	0.013 (0.216)	0.011 (0.218)	0.011 (0.215)	0.011 (0.215)	1	0.033 (0.219)	-0.027 (0.22)	0.006 (0.215)	0.009 (0.219)	0.019 (0.217)	0.044 (0.22)	-0.034 (0.216)	-0.022 (0.216)	0.009 (0.221)	0.022 (0.216)	-0.021 (0.219)	-0.008 (0.215)	0.009 (0.217)
(6)	-0.001 (0.216)	0.016 (0.204)	0.036 (0.208)	-0.144 (0.21)	-0.013 (0.217)	1	-0.09 (0.211)	0.015 (0.215)	0.012 (0.215)	-0.009 (0.214)	0.078 (0.223)	-0.157 (0.217)	-0.023 (0.208)	-0.015 (0.217)	0.034 (0.215)	0.026 (0.214)	0.036 (0.206)	0.045 (0.214)
(7)	-0.002 (0.216)	-0.012 (0.211)	0.041 (0.211)	-0.021 (0.211)	0.017 (0.217)	-0.07 (0.212)	1	0.01 (0.217)	-0.021 (0.214)	-0.063 (0.206)	-0.013 (0.214)	0.198 (0.192)	0.118 (0.188)	-0.035 (0.215)	0.014 (0.208)	-0.001 (0.209)	0.006 (0.192)	0.012 (0.204)
(8)	0.001 (0.218)	-0.005 (0.213)	0 (0.219)	-0.026 (0.218)	-0.005 (0.219)	0.047 (0.219)	-0.003 (0.22)	1	-0.01 (0.217)	-0.001 (0.217)	0.007 (0.218)	-0.005 (0.215)	-0.009 (0.215)	-0.011 (0.219)	-0.004 (0.217)	0.023 (0.22)	0 (0.216)	0.011 (0.217)
(9)	-0.011 (0.217)	-0.017 (0.209)	-0.023 (0.212)	0.051 (0.212)	-0.013 (0.218)	0.007 (0.213)	-0.046 (0.217)	0.033 (0.219)	1	0.013 (0.217)	0.005 (0.218)	-0.019 (0.214)	0.003 (0.217)	-0.005 (0.219)	-0.016 (0.217)	-0.012 (0.22)	0.004 (0.216)	-0.005 (0.217)
(10)	0.004 (0.216)	-0.115 (0.218)	-0.027 (0.212)	0.034 (0.216)	-0.013 (0.217)	0.024 (0.212)	-0.004 (0.214)	0.016 (0.217)	0.041 (0.216)	1	0.009 (0.214)	0.021 (0.208)	-0.105 (0.21)	0.052 (0.221)	0.04 (0.214)	-0.022 (0.214)	0.038 (0.208)	-0.006 (0.212)
(11)	0.011 (0.217)	0.084 (0.217)	-0.03 (0.213)	0.037 (0.216)	-0.005 (0.22)	-0.035 (0.213)	0.017 (0.217)	-0.008 (0.218)	0.013 (0.217)	-0.008 (0.217)	1	-0.076 (0.211)	0.092 (0.216)	0.003 (0.22)	0.066 (0.221)	-0.003 (0.216)	-0.026 (0.213)	-0.035 (0.216)
(12)	-0.012 (0.218)	-0.091 (0.211)	0.025 (0.214)	0.063 (0.215)	-0.006 (0.217)	-0.1 (0.214)	0.068 (0.216)	-0.001 (0.217)	0.02 (0.214)	0.064 (0.222)	-0.012 (0.214)	1	0.055 (0.19)	0.001 (0.214)	-0.006 (0.207)	0.021 (0.207)	-0.084 (0.193)	0.074 (0.207)
(13)	-0.004 (0.218)	-0.02 (0.209)	0.107 (0.222)	-0.016 (0.213)	0.055 (0.221)	-0.018 (0.211)	0.045 (0.215)	-0.006 (0.216)	-0.039 (0.216)	0.016 (0.215)	0.005 (0.216)	-0.005 (0.214)	1	0.001 (0.214)	0.066 (0.207)	-0.011 (0.208)	-0.207 (0.187)	-0.158 (0.212)
(14)	-0.026 (0.218)	0.04 (0.213)	-0.008 (0.211)	0.038 (0.214)	0.023 (0.222)	-0.039 (0.213)	0.014 (0.215)	-0.011 (0.217)	-0.005 (0.214)	0.06 (0.22)	-0.003 (0.216)	0.064 (0.22)	-0.01 (0.215)	1	-0.001 (0.219)	0.006 (0.215)	0.011 (0.215)	-0.001 (0.216)
(15)	-0.026 (0.218)	0.019 (0.202)	-0.054 (0.207)	-0.072 (0.207)	-0.018 (0.217)	0.15 (0.21)	-0.065 (0.213)	0.006 (0.218)	0.009 (0.21)	0.045 (0.215)	0.04 (0.214)	-0.06 (0.21)	0.021 (0.21)	-0.038 (0.214)	1	-0.016 (0.215)	-0.027 (0.209)	-0.049 (0.215)
(16)	-0.004 (0.217)	0.007 (0.205)	0.046 (0.205)	0 (0.207)	0.007 (0.215)	0.097 (0.203)	0.066 (0.21)	0.018 (0.216)	-0.119 (0.218)	-0.048 (0.21)	-0.007 (0.213)	-0.013 (0.213)	0.014 (0.212)	-0.04 (0.215)	0.048 (0.204)	1	-0.026 (0.212)	0.032 (0.216)
(17)	-0.009 (0.217)	-0.013 (0.198)	0.058 (0.202)	0.012 (0.2)	-0.038 (0.218)	0.044 (0.201)	0.068 (0.209)	-0.005 (0.218)	0.025 (0.21)	-0.057 (0.212)	0.017 (0.211)	-0.017 (0.206)	-0.021 (0.208)	-0.08 (0.213)	-0.06 (0.199)	0.11 (0.2)	1	0.089 (0.21)
(18)	0.046 (0.217)	0.042 (0.196)	-0.03 (0.198)	-0.07 (0.199)	0.004 (0.217)	0.278 (0.197)	-0.078 (0.206)	0.024 (0.215)	0.044 (0.21)	0.017 (0.207)	-0.085 (0.214)	-0.043 (0.207)	-0.075 (0.209)	-0.076 (0.212)	0.052 (0.197)	-0.063 (0.196)	0.08 (0.19)	1

Supplementary Table S5. Correlations of individual-level random effects across behaviors obtained by Model_ihF. The reported means are from the posterior samples (standard deviation in parentheses). Parameters in bold represent estimates whose 95% credible intervals do not include zero. The bottom half of the matrix depicts correlations of random effects from initiators. The top half of the matrix details correlations of random effects from recipients.

	Intercept	Initiator			Recipient			Same
		Age	Sex	Age:Sex	Age	Sex	Age:Sex	Household
(1) Comforting	**-1.90 (0.35)**	**0.69 (0.22)**	0.42 (0.31)	0.50 (0.29)	**-1.30 (0.26)**	-0.66 (0.49)	**-0.99 (0.36)**	0.58 (0.30)
(2) Dominating	**1.85 (0.15)**	**0.51 (0.13)**	-0.08 (0.19)	0.21 (0.18)	**-0.41 (0.13)**	0.29 (0.19)	0.01 (0.18)	-0.21 (0.19)
(3) Helping	**0.81 (0.17)**	**0.40 (0.15)**	0.37 (0.22)	0.20 (0.20)	-0.11 (0.14)	0.29 (0.21)	-0.02 (0.20)	**0.48 (0.20)**
(4) Leading	**2.52 (0.14)**	**0.61 (0.13)**	0.23 (0.18)	0.23 (0.17)	-0.13 (0.12)	0.29 (0.18)	0.04 (0.18)	-0.19 (0.18)
(5) Dirty Looks	**-1.06 (0.28)**	0.18 (0.26)	**1.47 (0.33)**	0.24 (0.31)	0.05 (0.21)	-0.07 (0.30)	0.16 (0.30)	**-1.06 (0.39)**
(6) Physical Aggression	**1.66 (0.15)**	0.09 (0.13)	-0.36 (0.21)	0.10 (0.19)	**-0.36 (0.13)**	0.00 (0.20)	0.14 (0.20)	-0.17 (0.20)
(7) Requesting for Access	**0.51 (0.21)**	-0.03 (0.16)	0.16 (0.24)	0.15 (0.22)	0.31 (0.17)	**0.49 (0.25)**	0.43 (0.23)	**-0.68 (0.24)**
(8) Requesting for Comfort	**-3.77 (0.53)**	**-1.34 (0.32)**	**-1.39 (0.66)**	-0.75 (0.41)	0.24 (0.31)	0.63 (0.44)	**0.85 (0.40)**	**1.46 (0.40)**
(9) Requesting for Help	-0.27 (0.22)	-0.13 (0.17)	-0.15 (0.27)	-0.07 (0.24)	**0.45 (0.17)**	0.43 (0.26)	0.22 (0.24)	**0.63 (0.24)**
(10) Requesting for Sharing	**0.95 (0.17)**	0.23 (0.14)	-0.05 (0.21)	-0.09 (0.20)	0.04 (0.14)	0.35 (0.21)	0.09 (0.20)	0.14 (0.21)
(11) Scolding	**1.57 (0.15)**	**0.50 (0.13)**	**0.53 (0.19)**	0.29 (0.18)	-0.03 (0.13)	0.19 (0.19)	0.12 (0.18)	-0.26 (0.19)
(12) Sharing	**0.63 (0.18)**	0.11 (0.15)	0.30 (0.22)	0.37 (0.21)	0.08 (0.15)	**0.45 (0.22)**	-0.33 (0.21)	0.06 (0.22)
(13) Supporting Opinions	0.08 (0.22)	**0.44 (0.17)**	0.15 (0.25)	-0.07 (0.23)	**0.37 (0.18)**	**0.62 (0.26)**	0.05 (0.25)	-0.50 (0.26)
(14) Taking Away	**0.86 (0.17)**	0.19 (0.15)	-0.13 (0.22)	0.05 (0.20)	-0.05 (0.14)	0.33 (0.21)	0.01 (0.20)	0.30 (0.21)
(15) Tattling	**0.52 (0.19)**	0.05 (0.15)	**0.74 (0.23)**	**0.50 (0.21)**	0.12 (0.15)	-0.06 (0.22)	-0.38 (0.21)	0.17 (0.22)
(16) Aggressive Teasing	**1.40 (0.16)**	**0.28 (0.14)**	**-0.44 (0.22)**	-0.07 (0.20)	**-0.28 (0.14)**	**0.43 (0.21)**	0.20 (0.20)	**-0.63 (0.22)**
(17) Playful Teasing	**2.06 (0.15)**	**0.48 (0.13)**	-0.10 (0.19)	0.02 (0.18)	-0.06 (0.13)	0.34 (0.19)	0.17 (0.18)	-0.22 (0.19)
(18) Verbal Aggression	**1.15 (0.18)**	**0.41 (0.15)**	**-0.72 (0.25)**	-0.43 (0.22)	0.04 (0.14)	0.28 (0.22)	0.11 (0.21)	**-0.64 (0.24)**

Supplementary Table S6. Posterior means (standard deviations in parentheses) of fixed effects in Model_iF. Parameters in bold represent estimates whose 95% credible intervals do not include zero.

	Intercept	Initiator			Recipient			Same
		Age	Sex	Age:Sex	Age	Sex	Age:Sex	Household
(1) Comforting	**-1.96 (0.36)**	**0.70 (0.22)**	0.40 (0.32)	0.49 (0.30)	**-1.31 (0.26)**	-0.66 (0.50)	**-1.00 (0.36)**	0.60 (0.31)
(2) Dominating	**1.85 (0.15)**	**0.52 (0.13)**	-0.09 (0.19)	0.21 (0.18)	**-0.42 (0.13)**	0.27 (0.19)	0.03 (0.18)	-0.23 (0.20)
(3) Helping	**0.80 (0.18)**	**0.39 (0.15)**	0.37 (0.22)	0.20 (0.20)	-0.12 (0.14)	0.29 (0.21)	-0.01 (0.20)	**0.48 (0.21)**
(4) Leading	**2.55 (0.14)**	**0.62 (0.12)**	0.23 (0.18)	0.22 (0.17)	-0.12 (0.12)	0.30 (0.18)	0.04 (0.17)	-0.19 (0.18)
(5) Dirty Looks	**-1.17 (0.3)**	0.17 (0.26)	**1.46 (0.34)**	0.26 (0.32)	0.04 (0.22)	-0.08 (0.31)	0.15 (0.31)	**-1.06 (0.39)**
(6) Physical Aggression	**1.65 (0.16)**	0.09 (0.13)	-0.36 (0.21)	0.09 (0.19)	**-0.37 (0.13)**	0.01 (0.20)	0.15 (0.19)	-0.18 (0.20)
(7) Requesting for Access	**0.48 (0.22)**	-0.05 (0.16)	0.14 (0.24)	0.20 (0.22)	**0.33 (0.17)**	**0.49 (0.24)**	0.39 (0.23)	**-0.71 (0.25)**
(8) Requesting for Comfort	**-3.83 (0.53)**	**-1.36 (0.33)**	**-1.42 (0.67)**	-0.78 (0.41)	0.23 (0.31)	0.60 (0.45)	**0.87 (0.39)**	**1.47 (0.41)**
(9) Requesting for Help	-0.30 (0.24)	-0.13 (0.17)	-0.15 (0.27)	-0.05 (0.24)	**0.46 (0.18)**	0.43 (0.26)	0.20 (0.24)	**0.63 (0.25)**
(10) Requesting for Sharing	**0.94 (0.17)**	0.23 (0.14)	-0.05 (0.22)	-0.08 (0.20)	0.04 (0.14)	0.34 (0.21)	0.10 (0.20)	0.14 (0.21)
(11) Scolding	**1.55 (0.15)**	**0.51 (0.13)**	**0.53 (0.19)**	0.28 (0.18)	-0.04 (0.13)	0.19 (0.19)	0.12 (0.18)	-0.28 (0.20)
(12) Sharing	**0.63 (0.19)**	0.10 (0.15)	0.31 (0.22)	0.39 (0.21)	0.07 (0.15)	**0.44 (0.22)**	-0.33 (0.21)	0.06 (0.22)
(13) Supporting Opinions	0.06 (0.23)	**0.44 (0.17)**	0.15 (0.25)	-0.06 (0.23)	**0.36 (0.18)**	**0.63 (0.27)**	0.06 (0.25)	**-0.52 (0.27)**
(14) Taking Away	**0.82 (0.18)**	0.18 (0.14)	-0.10 (0.22)	0.04 (0.20)	-0.06 (0.14)	0.28 (0.21)	0.02 (0.20)	0.29 (0.22)
(15) Tattling	**0.52 (0.19)**	0.05 (0.15)	**0.74 (0.23)**	**0.50 (0.21)**	0.12 (0.15)	-0.07 (0.22)	-0.36 (0.21)	0.18 (0.22)
(16) Aggressive Teasing	**1.39 (0.16)**	**0.29 (0.14)**	**-0.45 (0.22)**	-0.07 (0.20)	**-0.28 (0.14)**	**0.43 (0.21)**	0.20 (0.20)	**-0.63 (0.22)**
(17) Playful Teasing	**2.06 (0.15)**	**0.48 (0.13)**	-0.10 (0.19)	0.02 (0.18)	-0.06 (0.13)	0.33 (0.19)	0.18 (0.18)	-0.23 (0.19)
(18) Verbal Aggression	**1.13 (0.19)**	**0.42 (0.15)**	**-0.70 (0.25)**	**-0.44 (0.23)**	0.04 (0.14)	0.29 (0.22)	0.11 (0.21)	**-0.64 (0.24)**

Supplementary Table S7. Posterior means (standard deviations in parentheses) of fixed effects in Model_ihF. Parameters in bold represent estimates whose 95% credible intervals do not include zero.

		Trend of predicted probability with increasing initiator's age				Trend of predicted probability with increasing recipient's age			
		Same Household		Different Household		Same Household		Different Household	
		Female initiator	Male initiator	Female initiator	Male initiator	Female recipient	Male recipient	Female recipient	Male recipient
(1)	Comforting	Increasing	Increasing	Increasing	Increasing	Decreasing	Decreasing	Decreasing	Decreasing
(2)	Dominating	Increasing	Increasing	Increasing	Increasing	(1,2)	Decreasing	(0,1]	Decreasing
(3)	Helping	8	(8,10)	8	(7,9)	[3,4]	[4,6]	[2,3]	(4,7)
(4)	Leading	Increasing	Increasing	Increasing	Increasing	(4,5)	(3,5)	(3,4)	(3,6)
(5)	Dirty Looks	[3,4]	Decreasing	[3,4]	Decreasing	Increasing	Increasing	Increasing	Increasing
(6)	Physical aggression	[2,3)	(1,2)	(1,2)	Decreasing	[2,3]	Decreasing	2	Decreasing
(7)	Requesting for Access	2	Decreasing	1	Decreasing	Increasing	Increasing	Increasing	Increasing
(8)	Requesting for Comfort	Decreasing	Decreasing	Decreasing	Decreasing	Increasing	Increasing	Increasing	Increasing
(9)	Requesting for Help	Decreasing	Decreasing	Decreasing	Decreasing	Increasing	Increasing	Increasing	Increasing
(10)	Requesting for Sharing	[2,3)	(3,5)	[1,2)	Decreasing	10	Increasing	10	Increasing
(11)	Scolding	Increasing	Increasing	Increasing	Increasing	(9,10)	(9,10)	(9,10)	(9,10)
(12)	Sharing	5	(1,2)	(5,6)	Decreasing	(2,3)	Increasing	(1,2)	Increasing
(13)	Supporting Opinions	[3,4)	Increasing	[2,3)	Increasing	Increasing	Increasing	Increasing	Increasing
(14)	Taking Away	[2,3)	(2,3)	(1,2)	Decreasing	(5,6)	(8,9)	(5,6)	(9,10)
(15)	Tattling	[6,7]	[0,1]	[6,7]	Decreasing	[2,3)	Increasing	(1,2)	Increasing
(16)	Aggressive Teasing	(2,3)	(3,5)	(1,2)	(2,4)	[4,5]	Decreasing	[4,5]	Decreasing
(17)	Playful Teasing	5	Increasing	5	Increasing	[9,10]	[6,8]	[9,10]	[7,9]
(18)	Verbal aggression	(1,2)	Increasing	Decreasing	Increasing	Increasing	Increasing	Increasing	Increasing
(19)	Ownership Assertion	(1,2)	Decreasing	Decreasing	Decreasing	(6,8)	Increasing	(6,8)	Increasing

Supplementary Table S8. The trends of predicted probabilities of 19 behaviors with increasing ages of initiator and recipient, when the sexes and household status differed. The trends are summarized from Supplementary Fig. S8 and Supplementary Fig. S9 for initiator's age and recipient's age, respectively. For behaviors without a monotonic trend in the probability, we listed the age or age range (in years) of local maximum probability.

		Inidividual-level random effects				Household-level random effects			
		Initiator		Recipient		Initiator		Recipient	
		Model_ih	Model_ihF	Model_ih	Model_ihF	Model_ih	Model_ihF	Model_ih	Model_ihF
(1)	Comforting	0.62 (0.27)	0.21 (0.16)	2.07 (0.28)	0.35 (0.25)	0.20 (0.15)	0.23 (0.17)	0.25 (0.20)	0.28 (0.20)
(2)	Dominating	0.45 (0.08)	0.24 (0.10)	0.38 (0.08)	0.14 (0.09)	0.09 (0.07)	0.09 (0.07)	0.10 (0.08)	0.11 (0.08)
(3)	Helping	0.27 (0.15)	0.32 (0.15)	0.25 (0.13)	0.23 (0.13)	0.24 (0.13)	0.24 (0.13)	0.09 (0.07)	0.10 (0.08)
(4)	Leading	0.44 (0.07)	0.18 (0.09)	0.23 (0.07)	0.24 (0.07)	0.13 (0.08)	0.18 (0.07)	0.11 (0.07)	0.11 (0.07)
(5)	Look	0.40 (0.27)	0.23 (0.18)	0.20 (0.16)	0.19 (0.15)	0.62 (0.27)	0.46 (0.24)	0.21 (0.16)	0.19 (0.15)
(6)	Physical aggression	0.45 (0.10)	0.30 (0.12)	0.38 (0.09)	0.18 (0.11)	0.15 (0.10)	0.18 (0.11)	0.13 (0.09)	0.14 (0.09)
(7)	Requesting for Access	0.34 (0.17)	0.27 (0.16)	0.78 (0.14)	0.52 (0.16)	0.50 (0.16)	0.51 (0.14)	0.27 (0.17)	0.27 (0.18)
(8)	Requesting for Comfort	2.67 (0.40)	0.39 (0.27)	0.38 (0.29)	0.26 (0.21)	0.35 (0.29)	0.30 (0.23)	0.24 (0.19)	0.26 (0.20)
(9)	Requesting for Help	0.80 (0.20)	0.31 (0.20)	0.45 (0.22)	0.20 (0.15)	0.18 (0.14)	0.22 (0.15)	0.40 (0.19)	0.41 (0.19)
(10)	Requesting for Sharing	0.24 (0.13)	0.20 (0.13)	0.26 (0.14)	0.25 (0.14)	0.17 (0.11)	0.16 (0.10)	0.12 (0.08)	0.12 (0.09)
(11)	Scolding	0.49 (0.09)	0.14 (0.09)	0.17 (0.10)	0.13 (0.08)	0.14 (0.09)	0.15 (0.09)	0.13 (0.09)	0.13 (0.09)
(12)	Sharing	0.27 (0.14)	0.23 (0.14)	0.45 (0.13)	0.42 (0.13)	0.21 (0.12)	0.25 (0.13)	0.14 (0.10)	0.15 (0.11)
(13)	Supporting Opinions	0.26 (0.17)	0.27 (0.17)	0.71 (0.14)	0.59 (0.16)	0.21 (0.14)	0.18 (0.13)	0.17 (0.13)	0.21 (0.15)
(14)	Taking	0.21 (0.14)	0.20 (0.14)	0.11 (0.09)	0.12 (0.09)	0.34 (0.13)	0.33 (0.14)	0.28 (0.13)	0.30 (0.14)
(15)	Tattling	0.56 (0.14)	0.40 (0.16)	0.27 (0.15)	0.24 (0.14)	0.15 (0.11)	0.14 (0.10)	0.14 (0.10)	0.14 (0.10)
(16)	Teasing (aggressive)	0.44 (0.11)	0.33 (0.12)	0.26 (0.13)	0.20 (0.13)	0.12 (0.09)	0.13 (0.09)	0.14 (0.09)	0.13 (0.09)
(17)	Teasing (playful)	0.36 (0.09)	0.31 (0.09)	0.30 (0.09)	0.29 (0.09)	0.11 (0.07)	0.11 (0.07)	0.11 (0.07)	0.10 (0.07)
(18)	Verbal aggression	0.70 (0.13)	0.53 (0.13)	0.31 (0.16)	0.27 (0.15)	0.23 (0.15)	0.18 (0.13)	0.18 (0.12)	0.16 (0.11)

Supplementary Table S9. Variance estimates of the individual-level and household-level random effects in Model_ih and Model_ihF. The reported quantities are the standard deviations of the random effects, while the values in parentheses are the standard deviations of these quantities in the posterior samples.
